# Reconstructing Boolean network ensembles from single-cell data for unraveling dynamics in the aging of human hematopoietic stem cells

**DOI:** 10.1016/j.csbj.2021.09.012

**Published:** 2021-09-15

**Authors:** Julian D. Schwab, Nensi Ikonomi, Silke D. Werle, Felix M. Weidner, Hartmut Geiger, Hans A. Kestler

**Affiliations:** a Institute of Medical Systems Biology, Ulm University, Albert-Einstein-Allee 11, Ulm 89081, Germany; b Institute of Molecular Medicine, Ulm University, Albert-Einstein-Allee 11, Ulm 89081, Germany

**Keywords:** Single-cell network reconstruction, Boolean network ensembles, Ensemble dynamic analyses, HSC, Aging, NF-*κ*B, Single-cell RNA sequencing

## Abstract

Regulatory dependencies in molecular networks are the basis of dynamic behaviors affecting the phenotypical landscape. With the advance of high throughput technologies, the detail of omics data has arrived at the single-cell level. Nevertheless, new strategies are required to reconstruct regulatory networks based on populations of single-cell data. Here, we present a new approach to generate populations of gene regulatory networks from single-cell RNA-sequencing (scRNA-seq) data. Our approach exploits the heterogeneity of single-cell populations to generate pseudo-timepoints. This allows for the first time to uncouple network reconstruction from a direct dependency on time series measurements. The generated time series are then fed to a combined reconstruction algorithm. The latter allows a fast and efficient reconstruction of ensembles of gene regulatory networks. Since this approach does not require knowledge on time-related trajectories, it allows us to model heterogeneous processes such as aging. Applying the approach to the aging-associated NF-*κ*B signaling pathway-based scRNA-seq data of human hematopoietic stem cells (HSCs), we were able to reconstruct eight ensembles, and evaluate their dynamic behavior. Moreover, we propose a strategy to evaluate the resulting attractor patterns. Interaction graph-based features and dynamic investigations of our model ensembles provide a new perspective on the heterogeneity and mechanisms related to human HSCs aging.

## Graphical abstract

**Figure:**
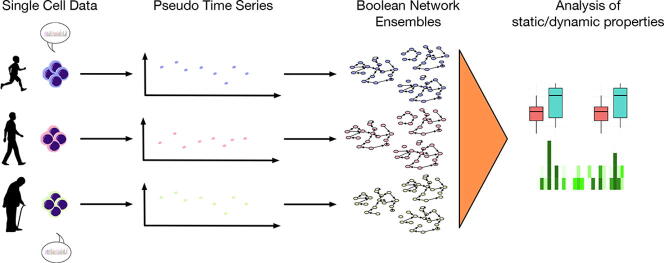

## 1 Introduction

Technologies that analyze regulatory dependencies in biological high throughput data support the notion that cellular decisions and aging are based on complex molecular networks [1]. Systems biology addresses the understanding of these complex interactions by proposing a holistic view of such complex regulatory processes. Central problems in single-cell-omics analyses are the identification of the underlying regulatory networks and modeling of their dynamic behavior over time.

For modeling dynamic behaviors, multiple dynamic models have been suggested, ranging from Boolean networks [2] to probabilistic rule-based models [3], to differential equation-based models [4]. By describing regulatory interactions through binary activity levels (0/FALSE/inactive, 1/TRUE/active), Boolean network models do not require kinetic parameters, which are often not available in biological studies [2]. Therefore, Boolean network models are very versatile and frequently applied for inferring and modeling gene regulatory networks.

Depending on the available type of data, Boolean networks can be inferred either by intense literature search [5], [6], [7], [8] or by reconstruction from time series data [9], [10], [11]. The literature-based inference has the advantage of combining different levels of information from expression, to protein interactions, to phenotypic traits [12]. Nevertheless, the reproducibility of curated models has been questioned due to the possibility of expert-biased knowledge [13]. On the other hand, the reconstruction of regulatory networks from expression data relies on algorithms able to reconstruct regulatory dependencies based on time series. These data-driven approaches have generated more and more interest with the quick evolution of omics data and sequencing technologies. In particular single-cell RNA sequencing has given the unique possibility to explore phenotypes at the individual cellular level. While the perspective of reconstructing regulatory networks from single-cell data is appealing, these approaches have been limited so far to the reconstruction of developmental or differentiation phenotypes [14]. This is due to the dependency of current reconstruction algorithms on time-ordering [14]. Our new approach instead, by assuming the cell populations'ś intrinsic heterogeneity, allows for reconstruction of single-cell-derived networks without time-ordering constraints, which we applied to the study of HSC aging.

HSCs form all blood cells in a process termed hematopoiesis [15]. The isolation and *in vitro* maintenance of HSCs are still experimentally very challenging approaches [16], [17], [18], [19]. Therefore, results from *in silico* modeling are of particular interest in this field. To this end, single-cell reconstruction approaches to study the differentiation of HSCs have recently been proposed [20], [21], [22].

Upon aging, HSCs are increased in number, but their activity is highly heterogeneous and impaired. This impaired function of aged HSCs might influence the immune system (AAIR) and is likely to be associated with leukemia [15]. Currently, the aging of human HSCs is primarily described in phenotypic terminology, with a number of molecular hallmarks of HSC aging being characterized [23], [24]. These are metabolic activation, reactive oxygen species (ROS) production, impaired autophagy, loss of polarity, cellular senescence, telomere extension, and increased DNA damage [23]. All these hallmarks are known to be connected to inflammation. Here, the NF-*κ*B pathway is known to play a crucial role, but the main players and mechanisms regulating this process are still not well characterized [23]. In addition, most of the information on HSC physiology and aging comes from pooled murine data, whereas human data are still not widely explored (Table A1). Murine and human HSCs share common regulatory mechanisms, still some differences do exist [25]. Hence, the determination of underlying mechanisms and regulatory networks of aging of HSCs is critical to design approaches to attenuate the aging-related impaired function of HSCs and identify markers distinguishing between chronological and biological aging [1], [26].

In the present work, we focus on developing and analyzing a network reconstruction pipeline taking advantage of the emerging single-cell sequencing techniques. The latter was applied to investigate the NF-*κ*B pathway involved in human HSC aging. The network reconstruction is based on data from the recently published work from Ratliff and colleagues [27], analyzing HSCs from eight healthy human beings of different ages separated into two groups – young and aged. Based on the heterogeneity present in single-cell data and especially HSCs, which are known to have varying levels of activation [28], [29] (Fig. A1-), we were able to reconstruct specific regulatory networks for each individual. This was only possible by implementing a new concept to retrieve pseudotime series within a certain population of single-cell data. Next, we established a new hybrid pipeline to reconstruct ensembles of regulatory networks to boost the speed of reconstruction, which allowed us to reconstruct large networks. Our new reconstruction strategy, which we named “*filtered best-fit*”, combines the detection of a filtered selection of input candidates [30] which serves, then, as a preprocessing step for the exact algorithm from Lähdesmäki and colleagues (Best-Fit Extension algorithm) [31]. Interaction graph-based measures and dynamic analyses of the resulting population of networks (ensembles) representing different age states of human HSCs indicated significant differences in terms of structural connections, heterogeneity, and activity levels in the populations of networks of aged HSCs.

## 2 Materials and methods

### 2.1 Boolean network models

Boolean networks are dynamic mathematical models applied to describe biological regulatory processes. These models are defined as a set of n compounds $X=\left\{x_{1},x_{2},\cdots ,x_{n}\right\},x_{i}\in B$ whose regulatory dependencies are described in a set of Boolean transition functions ${\{f}_{1},\cdots ,f_{n}\},f_{i}:B^{n}\to B$. In these functions, regulatory interactions are summarized by logical operators. The activity of each compound *x_i_* is considered to be either active (1) or inactive (0). The state of a network at a specific point in time *t* is therefore determined by a vector $\vec{x}\left(t\right)=\left(x_{1}\left(t\right),\ldots ,x_{n}\left(t\right)\right)$ containing all assigned activities for each compound at that time point. The study of network dynamics over time can be performed based on different updating schemes – synchronous [2], asynchronous [32], and probabilistic [33] updating. Applying these updating schemes to Boolean functions creates state transitions and, thus, edges from vertex to vertex (each representing one particular state) in the state transition graph [12]. Here, we consider synchronous updating, which requires the least assumptions [12]. Under synchronous updates, the transitions from one state to the next one $\overset{\to }{x}\left(t\right)\mapsto \overset{\to }{x}\left(t+1\right)$ are executed by updating all regulatory functions *f_i_* at the same time. This transition is described as $x_{i}\left(t+1\right)=f_{i}\left(\vec{x}\left(t\right)\right)$. Taking into account that each compound in the network has only two possible assigned values, the total number of states scales to 2^n^, n being the total number of compounds in the network [2]. Due to this deterministic nature of the state space, under synchronous update schemes, the model will eventually enter a recurrent sequence of states called an attractor. Each network can have either single or multiple attractors, which in turn can have single (fixed points) or multiple states (cyclic attractors). Attractors depict the long-term behavior of the model, and they have been connected to biological phenotypes [34], [35], [36], [37], [38]. Among others, Boolean networks are frequently used to simulate biological experiments and, thus, screen, for example, for potential drug targets [39].

Boolean network models were simulated using the R-package BoolNet [40] and synchronous update strategy. For attractor search, the SAT-based search algorithm in the BoolNet package was used.

### 2.2 Single-cell RNA-sequencing data set

Model reconstruction was performed on the publicly available single-cell RNA sequencing data from Ratliff and colleagues [27] (NCBI Gene Expression Omnibus GSE138544). This data set contains 730 samples of isolated peripheral blood long-term HSCs (LT-HSCs) ($\mathrm{li}n^{-}CD34^{+}CD38^{-}CD45RA^{-}CD49f^{+}$) from four young (ages 19, 21, 37, 40) and four aged (ages 61, 66, 68, 70) human individuals (two males and two females per group) as suggested by Ratliff and colleagues [27]. The dataset contains 83 to 94 single-cell measurements per individual. Sequencing was performed on a NovaSeq6000. The available dataset shows log normalized counts per million [27]. The presented dataset was selected among other available datasets of single-cell RNA sequencing datasets (Table A1). It is, to the best of our knowledge, the only dataset with multiple non-pooled individuals currently publicly available.

### 2.3 Data preparation and expression analysis

To reconstruct Boolean network ensembles of the NF-*κ*B pathway, the gene symbols from the dataset were mapped to Entrez IDs using the R-package biomaRt [41]. Based on the KEGG database [42], 101 genes were extracted as belonging to the human NF-*κ*B signaling pathway (hsa04064). All selected genes were then binarized using the BASCA algorithm from the R-package BiTrinA [43], [44]. We used the BASC significance test to evaluate which genes were significantly binarized (FDR, p < 0.05). In accordance with these results, we used 96 genes for the reconstruction of Boolean network ensembles. Besides Boolean network reconstruction, we performed expression analysis to screen for clustering of expression data according to the age of the individuals. Hierarchical clustering of the samples was performed using the ComplexHeatmap (Version 2.4.3, [45]). In addition to that, we plotted the data t-Distributed Stochastic Neighbor Embedding (tSNE) using the Seurat R-package (Version 4.0.1, [46], [47]). Motif search was performed using the igraph R-package (Version 1.2.6, [48]).

### 2.4 Generation of pseudo-time series

After binarization, time series for the 96 binarizable genes were generated to proceed with the network reconstruction. Therefore, the state of each single-cell measurement is assumed to be a potential predecessor or successor of the state of each other single-cell measurement coming from the same individual. Consequently, it is possible to form a large number of tuples of predecessor and successor time steps X(*t*) and X(*t* + 1) by a combination of random single-cell measurements. Given a total amount of single-cell measurements (*s*), the number of couples of predecessor and successor states is $\left(\begin{array}{c} s\\ 2 \end{array}\right)$. This is multiplied by two as each state in a given tuple can act either as a predecessor state or successor state. Hence, the total number of possible tuples is $\left(\begin{array}{c} s\\ 2 \end{array}\right)\cdot 2$.

From the binarized expression data, 1000 tuples of data points were randomly drawn and used as time points (predecessor and successor state) to generate pseudo-time series of length two for reconstruction. This means, e.g. for individual young A (19 years old) having 94 measurements, we receive 4371 couples of single-cell measurements, resulting in 8742 possible tuples. Out of them, 1000 tuples are picked to reconstruct the Boolean functions. This procedure was repeated 20 times.

### 2.5 Reconstruction of Boolean networks from binarized time series

Monotonicity is a dominant pattern in biological functions [49]. The algorithm by Maucher et al. [30] infers regulatory dependencies from binary time series of data based on this monotonicity assumption. To infer these dependencies, the algorithm measures Pearson correlation between the different genes and the successive network states [30]. The algorithm identifies the input variables with a chosen minimum influence for each regulatory factor individually by measuring the Pearson correlation of each variable and the corresponding output value of the regulatory factor. If the correlation is above a specified threshold, the interaction is considered to be relevant. This is repeatedly done for each regulatory factor to infer the complete network dependencies [30].

While the algorithm by Maucher et al. [30] returns regulatory dependencies, the Best-Fit Extension algorithm by Lähdesmäki et al. [31] allows for the reconstruction of Boolean networks from time series of expression data. The *best-fit* approach tests all possible combinations of inputs for each Boolean function to fit the dynamics of the given time series as correctly as possible. In more detail, the algorithm screens for the subset X' of all genes *X* , with up to *k* inputs. To find Boolean functions which match the measured observations in the time series data corresponds to the consistency problem [50]. Consequently, a Boolean function that best separates true and false samples in the data is sought. In the Best-Fit Extension from Lähdesmäki and colleagues [31], this is done based on *partially defined Boolean functions pdBFs(T,F)*, with $T,F\in {\left\{0,1\right\}}^{k}$. These *pdBFs* describe the true and false observations in the given time series of binary data. Each tuple of predecessor X(t) and successor time point X(t + 1) is added to the *pdBF(T,F)* as follows [31]:

$$\begin{array}{c} T=\left\{X^{'}\left(t\right)\in {\left\{0,1\right\}}^{k}:X_{i}\left(t+1\right)=1\right\}\;and\\ F=\left\{X^{'}\left(t\right)\in {\left\{0,1\right\}}^{k}:X_{i}\left(t+1\right)=0\right\}. \end{array}$$

Next, the number of inconsistencies in the pdBF is measured by intersecting T and F $\left(\varepsilon =\left|T\cap F\right|\right)$. The algorithm then chooses the input combinations X' with the least error *ε*. In the final step, a Boolean function based on these inputs is created using truth tables. The truth table is filled by iterating through all examples s=T∪F at all time steps *j* as follows:

$$f_{i}^{j}=\left\{\begin{array}{c} 0\;if\;s\in F\wedge f_{i}^{j-1}=?\\ 1\;if\;s\in T\wedge f_{i}^{j-1}=?\\ {}*\;else \end{array}\right)$$

Here ? means undefined and * indicates a conflict. f^0^ is initialized as $f^{0}=\left(?,\ldots ,?\right)$.

### 2.6 Inference of ensemble networks

Based on the binarized pseudo-time series and the *filtered best-fit* approach, Boolean functions were reconstructed for each individual independently. For each set of single-cell data obtained from one individual, 1000 tuples of data points were randomly drawn and used as time points (predecessor and successor state) to generate pseudo-time series for reconstruction. Next, Boolean networks were reconstructed from these pseudo-time series. For each of the eight individuals, eight populations of networks (ensembles), comprising all reconstructed Boolean functions for each gene, were generated. For dynamic analysis, we sampled 100 Boolean networks from each ensemble by randomly choosing one of the potential Boolean functions suggested by the reconstruction algorithm. This procedure was repeated for 20 random picks of the 1000 tuples of pseudo-time points per individual, resulting in a total of 2000 networks per individual.

### 2.7 Comparison of the reconstruction new pipeline with the best-fit approach

To evaluate the proposed reconstruction pipeline, we compared its computation time and performance to the original Best-Fit Extension approach. We created random networks of different sizes (20, 40, 60 to 200 in steps of 20) with scale-free topology as ground truth networks using the BoolNet R-package. Next, we created time series of different lengths from each of the random networks. For the first measurements, the number of time steps was fixed to 20 (for each of 100 networks per size 20 to 200). For the second measurements, we created a time series of |V| + 10 time points (where |V| is the number of nodes in the network, see Berestovky and colleagues [9]). Based on these time series, we reconstructed Boolean networks and measured the reconstruction runtime. To assess the reconstruction quality, we compared the interactions found in the reconstructed networks to the corresponding original network and measured the sensitivity and specificity of this prediction.

### 2.8 Interaction graph-based analyses

To assess structural changes during aging, we compared the reconstructed networks of the young and aged groups as well as the structural changes of each individual on its own. Therefore, we calculated different properties based on the suggested interactions between the genes of the reconstructed Boolean networks by measuring:

1). The number (#) of compounds, which are unregulated and, thus, set to a constant value (0/1) in the Boolean networks **(fixed genes)**,
2). The number (#) of compounds that have connectivity of 0 and are, consequently, disconnected from the rest of the graph **(isolated genes)**,
3). The mean number (#) of incoming edges across the ensemble of networks **(mean input)**, and
4). The mean number (#) of potential regulatory functions which could be found for each compound in the reconstructed process **(mean functions)**.

In addition, network motifs were also investigated across all individuals and by age groups (young vs aged). To this end, feed-forward loops and bi-fan motifs (as defined in Alon [51]) were analyzed. These motifs were selected based on their biological impact in regulating the stability of the protein-protein interaction network [52], [53].

We measured the differences between the two age groups using the Wilcoxon signed-rank test. We further validated the inferred regulatory interactions across age groups as well as for each individual by comparing the existence of the found interactions in the ensembles of Boolean networks also in the STRING-DB [54]. To do so, two matrices were combined: the first considering consistency of regulatory interaction within the ensembles, the second considering the existence of a given interaction in the STRING-DB database [54]. Below, the establishment of both matrices and their combination in a trinary match matrix is described.

The ensemble adjacency matrix has an entry of 1 at the position (i,j) if the compound *i* is present in the regulatory function of the compound *j* and 0 otherwise. In the case of multiple equally accurate regulatory functions for the same compound, the union across all present interactions is taken. Furthermore, a union of interactions found across all 20 different reconstructions is taken. For analyzing each age group individually, all interactions occurring across the four corresponding individuals are taken into account.

Similarly, a binary matrix has been constructed from the data available in STRING-DB [54], with entries having a value of 1 if there exists an interaction and 0 otherwise. A match was assigned considering paths that are either direct or indirect via only one additional node. This analysis has been performed both considering all possible sources of evidence (including, e.g., text mining and co-expression of genes), as well as for a restriction to interactions that were either present in curated databases or that were experimentally validated.

These two binary matrices for the ensembles and the STRING-DB were then compared to obtain a matching matrix. Here, 1 indicates that there is a match between interactions present both in the Boolean network as well as in STRING-DB. If there is no interaction in the Boolean network, the match matrix has a 0 entry. An entry of −1 indicates a mismatch, meaning that the interaction in the Boolean network was not found in STRING-DB or only exists via more than one indirect node.

### 2.9 Dynamic analyses

For further analysis of the reconstructed ensembles of Boolean networks, we investigated its dynamic behavior by screening its attractor landscape. To do so, we performed an exhaustive attractor search on each of the networks in the network population (ensemble). All simulations were performed using the synchronous update strategy as previously applied in other model simulations describing complex pathway interactions [5], [55], [56], [57].

First, we studied the mean number of attractors which could be found across all networks in the different ensembles and the repeated runs, and their number of states.

Second, we investigated the distribution of gene activity through the different attractor patterns. Here, the availability of ensembles of networks allows analyzing probabilities of attractor patterns. We summarized the binary states of the different attractors of each network and normalized them by the total number of attractor states. This procedure yields the probabilities of each gene to be active within the complete attractor landscape. Next, we summed up these probabilities for all network simulations within one ensemble and the repeated runs and normalized again by ensemble size and the number of repeated runs. Finally, results show an average probability of genes to be active in the long-term behavior of the networks in each ensemble over the repeated reconstruction runs.

## 3 Results and discussion

### 3.1 Fitting the heterogeneity of the aging process with a tailored systems biology approach

The aging process of HSCs is suggested to be characterized by a dynamic and heterogeneous behavior. While common effects of aging-related dysfunctions have been identified, not all HSCs in the elderly are thought to present this loss of function, which results in a likely heterogeneity of individual aged HSCs in single-cell expression data. Likely reflecting this heterogeneity, hierarchical clustering and t-SNE procedures on single HSCs from our selected dataset were not successful. In particular, analyzing the expression data of the genes in the known aging-related pathway as NF-*κ*B [24], [58] did not show relevant clustering between young and aged cells (Figs. A1-A4). New tools and approaches are warranted to depict this heterogeneity in the aging of HSCs.

For this reason, we reconstructed populations of Boolean models from single-cell RNA sequencing data. By studying the behavior and characteristics of the ensemble of individual networks, we provide novel tools to capture the heterogeneity of the aging process of HSCs. In the following, we will present in detail the rationale of our approach, together with the major insights from the ensembles of Boolean networks.

### 3.2 Filtered reconstruction of Boolean network ensembles from time series of single-cell data

All data-based reconstruction algorithms largely depend on the number of data and especially the time points which are available for reconstruction. Typically, there is only a small number of time points, and the reconstruction potential is limited. In this approach, we take advantage of the emerging single-cell high-throughput data and propose a new strategy to increase the amount of available time series within a population of cells.

**Fig. 1:**
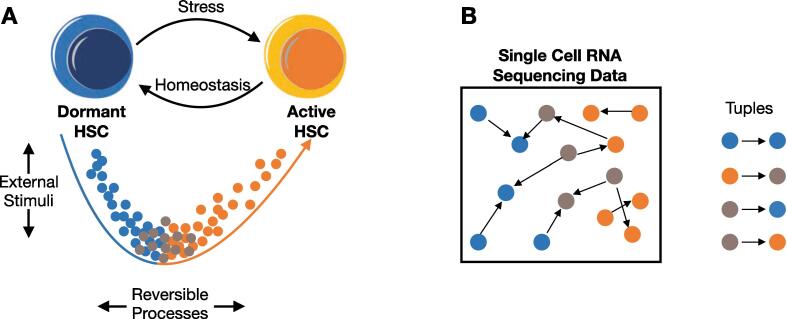
Heterogeneity of activity levels in the LT-HSC population. (A) Biological concept. Within the LT-HSC population, stem cells can reversely transit between more dormant or activated states without changing their stemness condition (no differentiation) [28], [29]. The heterogeneity of this population is influenced by environmental stimulation. (B) Generation of tuples from single-cell RNA sequencing data. Exploiting the heterogeneity of the LT-HSC population, pseudo-time-points assuming the possibility for each cell of the pool to switch to any of the other cell expression profiles. This implies that trajectories can be randomly selected within the population. Tuples were built by sets of randomly sampled trajectories from the expression data of each individual.

In single-cell sequencing data, though, each sample is described by a potentially large set of single-cell measurements. We group all measurements which belong to the same individual. Within these groups, we assume the state of each single-cell measurement is a potential predecessor or successor of the state of each other single-cell measurement in the same domain – each single-cell sample is treated as a pseudo-time-point and heterogeneity converted into pseudo-time-points. This assumption is based on experimental results showing that LT-HSCs can depict interchangeable activity states, implying that a population of LT-HSCs dynamically transits from one activity level to the other [28], [29] (Fig. 1A). Consequently, under this assumption, we can form a large number of tuples of predecessor and successor time steps X(t) and X(t + 1) by a combination of random single-cell measurements (see Fig. 1B and methods section 2.5).

Introducing this new concept for retrieving pseudo-time points from single-cell experiments leads to an increased number of time points compared to bulk sequencing experiments. This increase of information challenges the currently available reconstruction algorithms, impairing the efficient reconstruction of models.

**Fig. 2:**
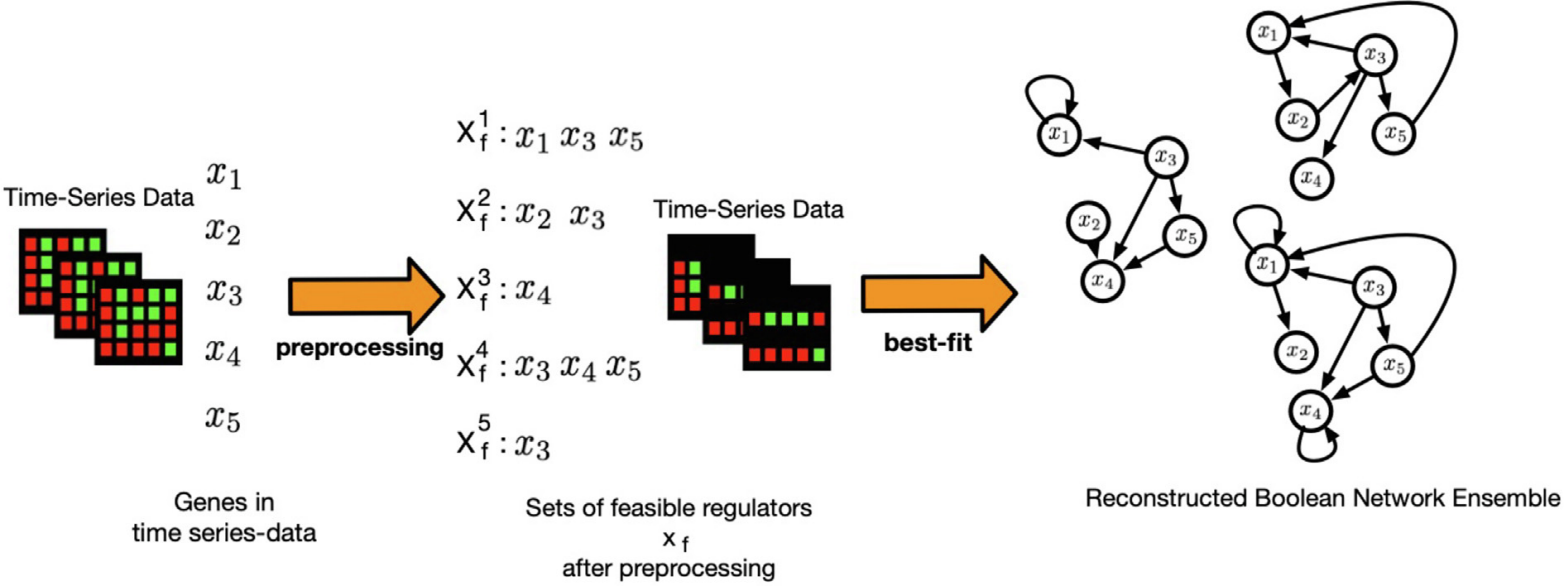
Overview of the new Boolean network reconstruction pipeline. The Boolean networks reconstruction pipeline combines a correlation-based approach to predict regulatory inputs by Maucher et al. [30] and the Best-Fit Extension algorithm by Lähdesmäki et al. [31]. The algorithm by Maucher et al. is used as preprocessing step to reduce the number of inputs that will be tested for the succeeding est-it xtension algorithm. This combination decreases the reconstruction time without losing meaningful results.

**Fig. 3:**
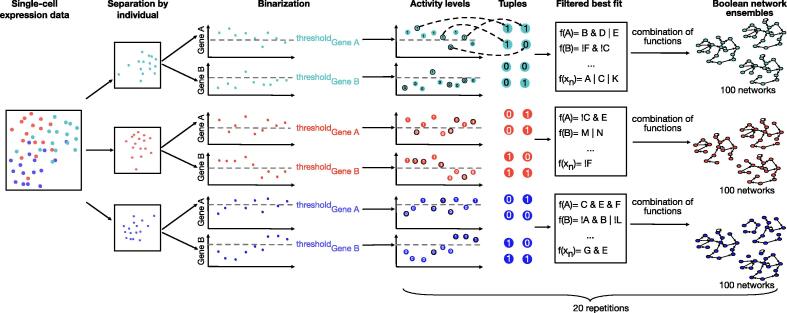
Schematic overview of the network reconstruction approach. In our approach, we first selected all single-cell samples from each group (red, blue, turquoise – here cell samples taken from the same individual). Next, data points are randomly sampled and ordered sequentially to create a series of pseudo-time-points for each group. This time series was then used for the reconstruction of Boolean network ensembles. The approach results in a set of Boolean network ensembles – one for each selected group. (For interpretation of the references to color in this figure legend, the reader is referred to the web version of this article.)

Boolean network reconstruction approaches need to be adapted to handle this data. We propose a new pipeline here that combines the search for potential regulatory inputs by Maucher et al. [30] and the Best-Fit-Extension algorithm [31] (Fig. 2). We use the approach by Maucher et al. [30] as a pre-processing step for the Best-Fit Extension by Lähdesmäki et al*.* [31]. In this reconstruction pipeline approach, the most feasible regulatory inputs $X_{\mathrm{fi}}\subseteq X$ for each regulatory input *x_i_* are determined using this approach [30]. In the next step, the Best-Fit Extension algorithm is used to reconstruct the corresponding Boolean functions with an input combination $X{'}_{l}\subseteq X_{fl}$ among the possible inputs *X_fi_* as derived from the pre-processing step (see Fig. 3). For the following reconstruction, we set the threshold for the preprocessing to 0.03 and the maximum number of inputs to five. Hence, instead of testing all possible input combinations in the reconstruction of Boolean networks, we only considered the ones selected by our preprocessing step. Based on the reconstruction results, we then create a population of Boolean networks (ensemble) by randomly sampling one regulatory function from the reconstructed set of potential functions for each compound of the modeled system.

In a first analysis, we compared the new pipeline to the original best-fit approach. To do so, we used randomly generated Boolean networks of different sizes, generated time series of data, and used those for the reconstruction of Boolean network ensembles. Reconstruction results were then compared to the original networks. Results showed, first, reducing the number of input combinations to test using the est-it xtension and, thus, a speed up the algorithm itself especially when considering noisy data (see Appendix Figs. A5 and A8). With an increasing number of time steps this effect becomes more prominent. Second, results also show an increased sensitivity when recapitulating the original networks based on the noisy data (see Appendix Figs. A6 and A9) while specificity of both approaches is in similar ranges (see Appendix A7 and A10).

### 3.3 Network populations (ensembles) reconstruction and structural properties in the process of aging

**Fig. 4:**
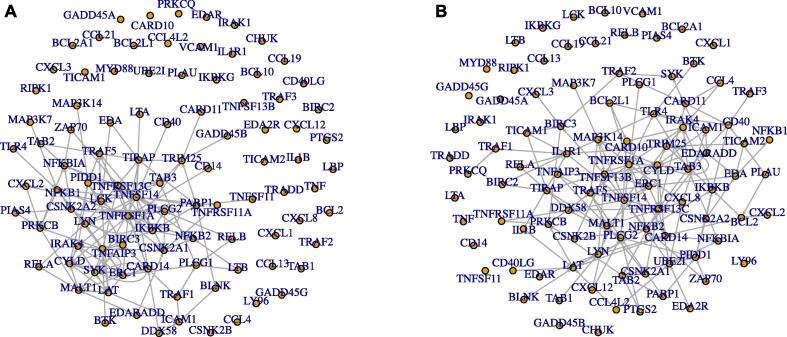
Interaction graph of one example network drawn randomly from the ensemble of one random individual from the young group of networks (A) and the aged group of networks (B).

**Fig. 5:**
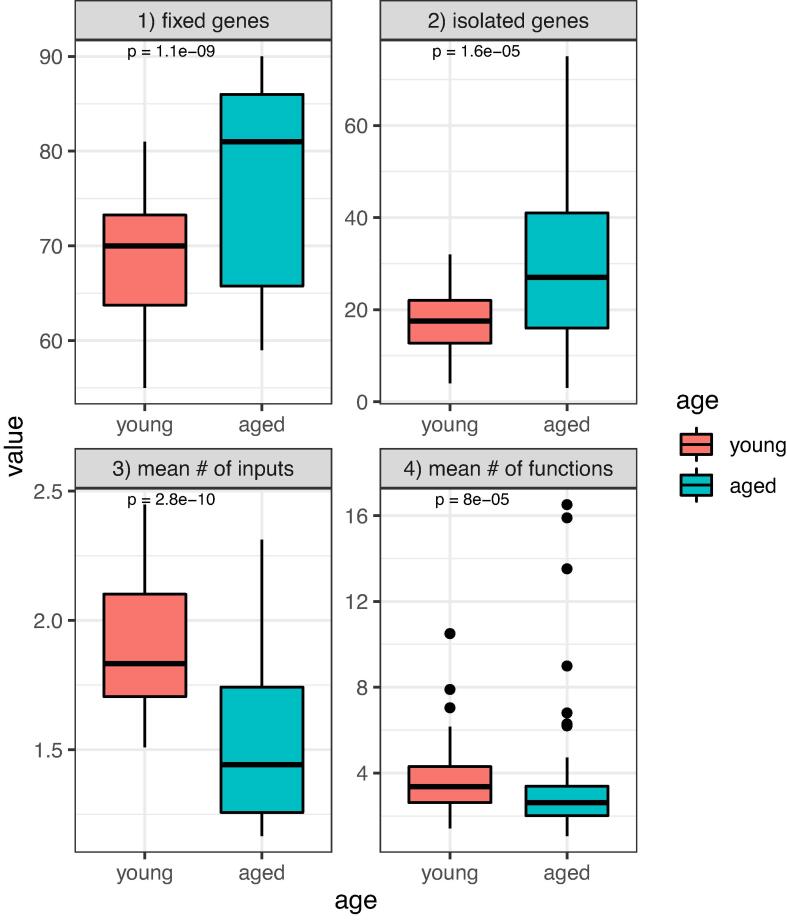
Structural properties of young and aged HSCs. We compared the static properties across the Boolean network ensembles of HSCs from all young and all aged individuals, respectively. Each panel describes one of the topological properties of the interaction graph of the underlying networks. 1) the number of fixed genes, 2) the number of isolated genes, 3) the mean number of regulatory inputs per gene per network in the ensemble, and 4) the mean number of reconstructed functions per gene per network in the ensemble. We measured the differences between the two age groups using Wilcoxon signed-rank test. Results for all measured properties show a significant difference (p < 0.05) between the two groups.

Using the described approach, we reconstructed ensembles of Boolean networks modeling the NF-*κ*B signaling pathway for each of the eight individuals. This revealed eight ensembles of Boolean networks – one representing each individual (exemplarily shown in Fig. 4). The reconstructed networks comprise up to 950 potential Boolean functions for one regulatory compound, depicted as a node in the network. The mean number of reconstructed Boolean functions per compound was between 1.06 and 16.50 across the different individuals and the 20 repeated runs. Reconstructed Boolean functions had between 1 and 5 regulatory inputs (Fig. 5 and Fig. A11). By comparing the reconstructed functions among young (19–40 years old) and aged (61–70 years old) individuals, we could show an increase in mean functions (p = 8·10^−5^) and regulatory inputs (p = 2.8·10^-10^) in the young phenotype compared to the aged individuals, while fixed genes (p = 1.1·10^−9^) and isolated ones (p = 1.6·10^−5^) were lower in the young reconstructed networks (for a precise definition of investigated groups of nodes see methods, section 2.6). These findings hint that in aged HSCs, regulatory interactions are muted, which would be consistent with a reduced response to stimulation as described by Schwab et al. [11]. We observed that the reconstructed networks of one individual (66 years old) had similar interaction graph-based features as the networks from the young group (Appendix Figs. A11 and A14), especially compared to the youngest individuals. This might indicate a younger biological age compared to the chronological one since this individual may rely on a network wiring with similar properties to the younger ones.

For further validation of the reconstructed Boolean functions, we compared the reconstructed interactions to interactions present in the STRING-DB database [54] (see Appendix Fig. A12). Considering all interactions within this database, we were able to validate 94.1% of reconstructed regulations for the young phenotype and 94.0% for the aged phenotype across all individuals in the respective group. Even applying stricter measures and only considering experiments and databases (see Methods, Section 2.8), we still found 66.1% of reconstructed regulations via direct interactions or interactions with one intermediary step for the young phenotype and 65.1% for the aged phenotype. These results further validate the reconstructed interactions. Some of the reconstructed regulations were not listed as direct interactions in STRING-DB. Our reconstruction approach might thus point to so far unknown interactions, although these will require confirmation by additional experiments.

**Fig. 6:**
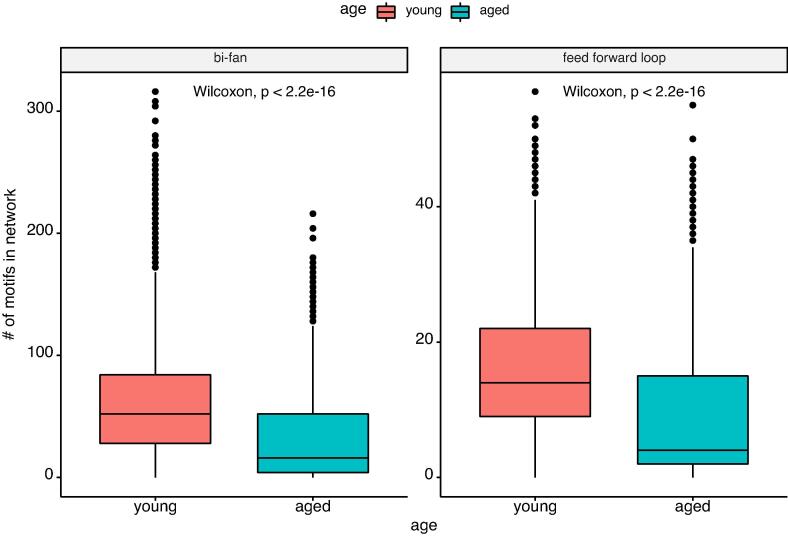
Boxplots showing the number of bi-fan and feed-forward loop motifs measured in 2000 randomly sampled networks from each individual’s ensemble (100 networks with 20 repetitions). Boxplots group the individuals by corresponding age group. We measured the differences between the two age groups using Wilcoxon signed-rank test and Bonferroni correction for multiple testing.

We performed screening of feed-forward loop and bi-fan motifs for each of the reconstructed networks. The number of each of these motifs for each network was computed and then used for comparison between the two age groups (see Fig. 6) and the eight individuals (see Fig. A14). Results show that the number of both motifs is significantly increased (Wilcoxon signed-rank test, p < 0.05) in the young group compared to the aged one.

### 3.4 Altered dynamics affecting aging

The human HSC aging process is still a widely unexplored field. In particular, still, a wide range of open questions are remaining on heterogeneity and its effects on biological aging in contrast to chronological aging. While being aware of the limited sample size of eight individuals, the results of the study might be a first attempt that justifies further exploration in this area in larger studies. Hence, we analyzed the dynamics of the group of Boolean networks by computing the attractor landscape. The dynamic analysis aims to get an initial understanding of general dynamics during aging in the NF-*κ*B pathway. Dynamic analyses have the potential to identify new drivers of aging in humans. In the following, we will deepen our analyses on the retrieved attractor landscape focusing on gene activities attempting to match the model behaviors to literature-reported aging activities.

**Fig. 7:**
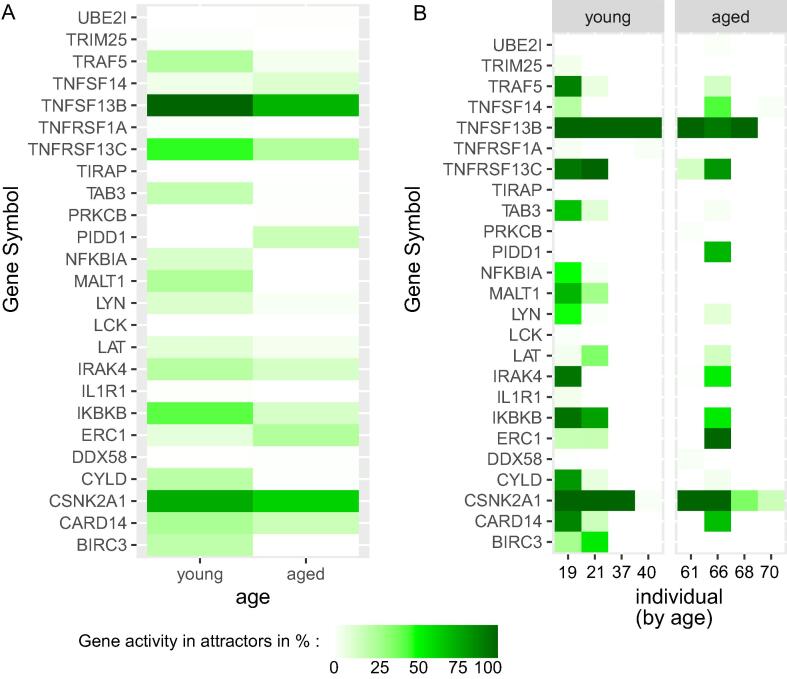
Changing activity levels in the process of aging. Figures showing the average activity of each gene in the attractor landscape of the simulated Boolean network ensembles. (A) The average activity of all non-zero genes over all ensembles of the young phenotype group (left column) and the aged phenotype group (right column) is depicted respectively. (B) The gene activity in the attractors of the ensembles averaged for each individual is shown. The individuals are sorted by increasing age (see x-axis). The color of the heatmap indicates the activity of a gene, ranging from 0% (white) to 100% (dark green). (For interpretation of the references to color in this figure legend, the reader is referred to the web version of this article.)

First, we studied the networks’ general long-term behavior. To do so, we evaluated both the number of attractors retrieved and their lengths (see Fig. A13). While a general tendency towards having few attractors with only one state is observed among young and aged networks, we observe an increase in the number of attractors (p = 1.10^−15^) and length ($p=1.5∙10^{-32}$) for the young ones. It is likely favorable for a regulatory network to have a majority of only single state attractors since they are considered to be more stable and less likely disturbed [12], [59]. This observation is consistent with a deep and stable quiescence of HSCs. In addition, Ikonomi et al. also retrieved a single state attractor describing the same HSC population in young physiological conditions [5]. The increase in the number of attractors in the network reconstructed for young HSCs might be linked to their ability to promptly respond to stimulation and which is reduced in aged HSCs [60]. Together with our results from the structural analysis (Fig. 5), our reconstructed networks in young HSCs show a larger number of connections and, therefore, hint at potential responses to sudden changes of external stimulations. Seeing these changes already in the LT-HSC population (for the NF-*κ*B pathway), which are considered to be majorly quiescent, also sustains the hypothesis that major dysregulations in hematopoiesis during aging arise from differentially prompted and reactive HSCs [23], [24]. Following this idea of a different intrinsic rewiring of the quiescent LT-HSCs, which prompt differential responses without *per se* causing a direct activation of the HSC, we deepened our analysis on the retrieved attractor landscape. To do so, we first analyzed the general activity tendencies between young and aged individuals and the inter-individual heterogeneity (Fig. 7). Second, we matched the activity of genes within the attractors to published phenotypes of young and aged HSCs (Table 1). The dynamic analysis intends to give initial insights into the general dynamics during aging in the NF-*κ*B pathway and second to identify potential new drivers of aging in humans. Together with these two points, literature validation of both changing and fixed gene activities in the attractor patterns aims at a first evaluation of the attractor patterns. To do so, we matched activities in the attractor to expected changes during HSCs aging or features expected from LT-HSCs. In this sense, our literature search revealed that over the 96 genes in our attractor patterns 78.73% could be matched with expected behaviors, while 12.5% of genes have no described function in HSCs (Tables A2 and A3). Finally, only 6.25% of genes did not match behaviors expected for HSCs from literature. As a general observation, most inactive genes were either related to leukemic transformation or to blood cell differentiation (see Table A3), which are indeed not likely to influence the activity of healthy HSCs.

**Table 1:** Activity of genes in the aging process.

Activity levels during aging young → aged	Compounds with altering in the Boolean network ensembles	Biological impact of altered activity	References
	BIRC3, IRAK4	Loss of rescue mechanisms for apoptosis causing impaired survival	[68], [69]
	CARD14, IKBKB, CYLD	Loss of quiescence	[70], [71], [72], [73], [74], [75], [76]
	TRAF5, LAT	Myeloid skewing	[77], [78], [79], [80]
	MALT1	Compensatory inhibition of NF-κB activation via induction of quiescence. Loss of NF-κB direct inhibition	[62], [61]
	NFKBIA	Propensity to increased inflammatory response via NF-κB activation	[81]
	LYN	Impaired repopulation potential	[82], [83]
	ERC1	Cellular polarity alteration causing reduced motility. Presence of ERC1 promotes the turnover of focal adhesions	[84], [85], [86], [87], [88]
	TNFRSSF13C	Impaired lymphoid specification	[89], [90]
	TNFSF13B	Stable immune response	[63], [64]
	CSNK2A1	Resistance to senescence	[65]
	PIDD1	Control of balance between repair and apoptosis after DNA-damage	[66], [91]
	TNFSF14	Loss of quiescence due to increased cycling	[92]

Next, in this use-case, we investigated the change of activities by comparing gene activity of the different age groups. This analysis aims to point out possible age-related activities which need to be validated also on larger data sets once they become available.

We observed the tendency of a decrease in activity levels within the attractors ranging from young to aged phenotypes (Fig. 7). This tendency was observed in both the overall averaged young and aged groups and for each individual. Interestingly, while this decrease in activity levels is preserved during aging, still a certain heterogeneity among individuals is observed (Fig. 7).

Along with the attractor patterns, we divided the gene activity into three subgroups: 1) decreased, 2) remaining stable, and 3) increased in aging (Table 1). IRAK4 and TNFRSSF13C, both downregulated in our attractors, were shown to be decreased in aged HSCs encouraging again the correctness of the ensembles retrieved dynamics. Most of the remaining genes in the first subgroup are studied in the context of loss of function of HSCs, but dysregulation of their activity has not yet been linked to HSC aging. A non-trivial example is MALT1 that was also observed to be decreased with aging in our attractors. This reduced activity would lead to a double-edged effect. On the one side, loss of MALT1 promotes HSC quiescence [61]. On the other side, it skews hematopoiesis towards myeloid cells [61]. As MALT1 is an inhibitor of NF-*κ*B, its loss induces the increase of the latter. Nevertheless, MALT1 loss also has a positive effect on quiescence [61]. This might counteract the increased stress-response induced by NF-*κ*B that normally leads to cell cycle entry [62].

Furthermore, few genes showed either a stable active behavior or even an increase in activity throughout the aging process (Table 1). The function of stably active genes could be linked to a stable immune response [86], [87] or resistance to senescence [65].

Finally, our results show that PIDD1 is only active in one aged individual (Aged A, 66 y.o.). Interestingly, the reconstructed networks of this individual already showed more similarity to interaction graph-based features and network motifs to the youngest individuals (19 and 21 y.o.) compared to the remaining individuals of the aged group (61, 68 and 70 y.o.). A potential explanation for that might relate to a discrepancy between biological and chronological age for this individual. Notably, literature research revealed that PIDD1 activity is connected to DNA damage response [66].

Following this hypothesis, we further deepened the literature screening looking for an explanatory mechanism on the potential implication of PIDD1 in delaying age-related processes. PIDD1 is suggested to act as a switch between DNA damage response and apoptosis by regulating the activation of first damage response via NF-κB, and in a second moment apoptosis via Caspase-2 [66]. While PIDD1 activity has still not been investigated in HSCs, the HSC phenotype of Caspase-2-deficient cells is known [67]. Caspase-2 deficient mice develop normally but show aging-related dysfunctions. In addition, when challenged by oncogenic stimuli or stress, Caspase-2 deficient mice show enhanced tumor development [67]. Overall, this supports the idea that a reactivation of PIDD1 could contribute to a delayed aging process, leaving these initial results open for further experimental validation.

Altogether our dynamic analyses indicate a certain level of heterogeneity in the behavior of our ensembles of models. This suggests that the description of aging does not follow the idea of “one fits all” but might come with different mechanisms and activities. This idea is reflected in the distinction between biological and chronological aging [1]. Interestingly, the Boolean network analyses of this dataset showed diverging properties among the different individuals. Even though these results come from a pool of eight human individuals, we found patterns in our network analyses which match general mechanisms and hallmarks known in LT-HSC maintenance and aging (Appendix Tables A2 and A3, and Table 1). Considering the novelty of the approach, the results point to interesting findings which encourage further studies and experimental validations in this direction.

## 4 Conclusion

Here, we present a new method to reconstruct data-driven ensembles of regulatory networks from single-cell RNA-sequencing data potentially applicable to a wide variety of sequencing data. Taking advantage of the intra-cell population heterogeneity, the approach exploits the generation of pseudo-time points of populations of the same cell type. These pseudo-time points enable the reconstruction of Boolean network ensembles. To handle such a potentially large (pseudo-) time series, we furthermore developed a new Boolean network reconstruction pipeline by adding a correlation-based screening of potential regulatory dependencies as a pre-processing step to the est-it xtension algorithm. The pre-processing step not only decreases computational time but also is more robust to noisy data.

Our use-case, the analysis of aging-related changes in NF-κB signaling based on human HSCs single-cell data could successfully capture the heterogeneity of the aging process. Using the network reconstruction approach enabled us to analyze structural and dynamic properties. By ordering the individuals along changes within these properties, we could find similar tendencies to their order by age which are not evident in the expression data analyses.

## CRediT authorship contribution statement

**Julian D. Schwab:** Conceptualization, Software, Methodology, Formal analysis, Data curation, Visualization, Writing - original draft, Writing - review & editing. **Nensi Ikonomi:** Conceptualization, Formal analysis, Investigation, Visualization, Writing - original draft, Writing - review & editing. **Silke D. Werle:** Conceptualization, Formal analysis, Investigation, Visualization, Writing - original draft, Writing - review & editing. **Felix M. Weidner:** Formal analysis, Software, Visualization, Writing - original draft, Writing - review & editing. **Harmut Geiger:** Formal analysis, Writing - review & editing. **Hans A. Kestler:** Project administration, Supervision, Funding acquisition, Conceptualization, Formal analysis, Writing - original draft, Writing - review & editing.

## Declaration of Competing Interest

The authors declare that they have no known competing financial interests or personal relationships that could have appeared to influence the work reported in this paper.
