# Supplementary material for: Reconstructing Boolean network ensembles from single-cell data for unraveling dynamics in the aging of human hematopoietic stem cells
Source: Comput Struct Biotechnol J. 2021 Sep 15;19:5321–32. doi: 10.1016/j.csbj.2021.09.012 (PMC8487005; doi:10.1016/j.csbj.2021.09.012)
Supplement: Supplementary data 1 [file mmc1.pdf]

Appendix

Reconstruction Boolean network ensembles form single-cell data for unravelling dynamics in the aging of human hematopoietic stem cells

Julian D. Schwab[a,#], Nensi Ikonomi[a], Silke D. Werle[a,#], Felix M. Weidner[a,#], Harmut Geiger[b], Hans A. Kestler[a,*]

[a]Institute of Medical Systems Biology, Ulm University, Albert-Einstein-Allee 11, 89081 Ulm, Germany
[b]Departement of Molecular Medicine, Ulm University, Albert-Einstein-Allee 11, 89081 Ulm, Germany
[#]equal contribution
*corresponding author

Tables

Table A.1 Available single-cell RNA sequencing data for HSC aging. Summary of available datasets including information on reference, species, number of samples, and details on the sample composition.

Author	Accession number	Species	#Samples	Comment
Kowalczyk et al. 2015 [1]	GSE59114	*Mus musculus*	2128	-Two mouse strains<br>-All mice are pooled<br>-LT-HSC, ST-HSCs, and MPPs from young and old mice
Grover et al. 2016 [2]	GSE70657	*Mus musculus*	135	-2 ages<br>-only LT-HSCs
Kirschner et al. 2017 [3]	GSE87687	*HPC7 cells from mus musculus*	12	-TPO treatment<br>-4 points in times of treatment

	GSE87631	*Mus musculus*	1152	-2 mouse strains: WT and Jak2V617F<br>-2 ages (young and old)<br>-mice are pooled
Ratliff et al. 2020 [4]	GSE138544	*Homo sapiens*	730	-8 individuals<br>-8 time points

Table A.2 Complete inactive genes in all ensemble networks. The color legend indicates if the literature findings match the expected behavior of a quiescent LT-HSC. Green indicates that the activity is coherent with quiescent HSC, while for pink genes, the activity is contrasting. For blue highlighted genes, we did not find any information in the context of HSCs. In total, genes that have an activity level < 1% are considered to be inactive.

Gene	Literature	Reference
ZAP70	T-, B-, NK cells specific factor. Its loss causes loss of CD8+.	[5, 6]
VCAM1	VCAM-1 is minimally expressed on most resting vascular endothelial cells and is inducible in many tissue vascular beds following injury or stress.	[7]
TRAF3	Evidence of activity in myeloid cells.	[8]
TRAF2	TRAF2 is both a target of CYLD and an activator of TNIK and CTNNB1. Loss of TNIK does not alter HSC but his hyperactivation causes leukemic transformation.	[9, 10]
TRADD	TRADD is activated upon stress stimulation.	[11]
TRAF1	Activated upon TPO specific stimulation.	[12, 13]
TNFSF11 (RANKL)	Involved in osteoclasts formation. Expressed by osteoblasts and stroll cells. It is an NF-κB activator that binds to its surface receptor in osteoblast and osteoclast progenitors.	[14]
TNFRSF1A	TNFRSF1A (p55) is constitutively expressed. Its loss does not affect HSCs.	[15]
TNFRSF11 A	Is the receptor of RANKL and is involved in osteoclast differentiation.	[14]
TNFAIP3	Its expression is reduced in aged HSC. Nevertheless, it is induced upon TNF stimulation.	[16, 17]
TNF	External stimulator of HSC behavior.	[17]

TLR4	Hematopoietic progenitors express TLR4. When stimulated with TLR4 ligand myeloid progenitors give rise to monocytes or macrophages.	[18, 19]
TIRAP	Constitutive expression of TIRAP in HSCPCs promotes upregulation of interferon-γ promoting bone marrow failure. It is also responsible for the induction of myelodysplastic syndrome.	[20]
TICAM2	Neutrophil influencing factor, whose activity relates to their exhaustion.	[18]
TICAM1	Responsible for response to bacterial-produced LPS in HSCs. Plays a role therefore in infections and stress-induced proliferation of HSCs.	[21]
TAB2	Tab1/Tab2 double deletions completely eliminated the reconstitution activity of HSCs, whereas Tab1 or Tab2 single deletion did not cause any abnormality. Tab1/Tab2 double deficient lineage-negative, Sca-1+, c-Kit+ (LSK) cells did not proliferate and underwent cell death. Relevant for HSC activation, nevertheless here we are looking at LT-HSCs, which are not activated yet. Nonetheless, mong bone marrow cells, the mRNA levels of Tak1, Tab1, and Tab2 were significantly higher in the undifferentiated populations including LT-HSCs and progenitor cells compared to differentiated bone marrow cells.	[22]
TAB1	Tab1/Tab2 double deletions completely eliminated the reconstitution activity of HSCs, whereas Tab1 or Tab2 single deletion did not cause any abnormality. Tab1/Tab2 double deficient lineage-negative, Sca-1+, c-Kit+ (LSK) cells did not proliferate and underwent cell death. Relevant for HSC activation, nevertheless here we are looking at LT-HSCs, which are not activated yet. Nonetheless, mong bone marrow cells, the mRNA levels of Tak1, Tab1, and Tab2 were significantly higher in the undifferentiated populations including LT-HSCs and progenitor cells compared to differentiated bone marrow cells.	[22]
SYK	Largely expressed in progenitors and Vascular endothelial cells. Expressed as fusion protein TEL-SYK causes fatal myelopoiesis in mice.	[22]
RIPK1	Hematopoietic RIPK1 deficiency results in bone marrow failure caused by apoptosis and RIPK3-mediated necroptosis.	[23]
RELA	RELA is down-regulated in LT-HSCs by ZFP521, which preserves their quiescence and self-renewal. Activation of RELA via loss of ZFP521 leads to	[24]

	uncontrolled NF-κB activation, loss of quiescence, increased proliferation, and apoptosis.	
RELB	Knockout induces excessive quiescence via loss of self-renewal and impaired engraftment of HSCs.	[25]
PTGS2	PTGS2 is relevant in HSC formation and recovery after injury. Its up-regulation promotes the expansion of HSCs.	[26, 27]
PRKCQ	Mainly expressed in muscle and lymphoid tissue (T and B cells, thymocytes). Its expression is connected to the oncogenic growth of stem cells.	[28, 29]
PRKCB	Hyper expressed in HSCs during psoriasis disease.	[28]
PLCG2	Specific to megakaryocytes.	[30]
PLCG1	Required for the maintenance of leukemic stem cells.	[31]
PLAU	PLAU is the receptor of uPAR. Mobilized HSCs are uPAR negative.	[32]
NFKB2	Constitutive activation of NFKB2 and 1 causes bone marrow failure and loss of quiescence gene signature.	[33]
PIAS4	-	-
PARP1	Involved in oxidative stress protection of HSC induced by salidroside. Induced in DNA damage response and highly expressed in AML, where it is used as a prognostic factor.	[34, 35]
NFKB1	Constitutive activation of NFKB2 and 1 causes bone marrow failure and loss of quiescence gene signature.	[33]
MYD88	Involved in myeloid and lymphoid differentiation. Activated in HSCs after bone marrow transplantation. Also known to drive the proliferation of HSCs upon infection.	[36, 37]
MAP3K7	Tak1 single or Tab1/Tab2 double deletions completely eliminated the reconstitution activity of HSCs. Among bone marrow cells, the mRNA levels of Tak1, Tab1, and Tab2 were significantly higher in the undifferentiated populations including LT-HSCs and progenitor cells compared to differentiated bone marrow cells.	[22]
MAP3K14	Constitutive activation of MAP3K14 (NIK) impairs self-renewal and induces bone marrow failure.	[38]
LY96	Leukocyte specifically expressed gene.	[39]
LTA	Lymphoid lineage marker that is expressed at low levels in HSCs and progenitors.	[40]
LTB	Lymphoid lineage marker that is expressed at low levels in HSCs and progenitors.	[40]
LCK	T cell-specific activated promoter.	[41]
LBP	Mediator of endotoxemia.	[42]

IRAK1	IRAK1 is overexpressed in cells with myelodysplastic syndromes.	[43]
IL1B	Important for differentiation into myeloid cells and chronic IL-1 exposure impairs HSC self-renewal capacity.	[44]
IKBKG	-	-
ICAM1	ICAM-1 is a secreted factor by the bone marrow and ICAM-1 deficient mice HSCs show impaired quiescence.	[45]
GADD45G	Can induce differentiation of LT-HSC and cytokine induction.	[46]
GADD45B	Important for the genotoxic stress response of the bone marrow and thus affect the survival of hematopoietic cells.	[47]
GADD45A	Important for the genotoxic stress response of the bone marrow and thus effect the survival of hematopoietic cells.	[47]
EDARADD	-	-
EDAR	-	-
EDA2R	Upregulated in the bone marrow post-irradiation.	[48]
EDA	-	-
DDX58	Induced during myelopoiesis.	[49]
CXCL8	Rapidly mobilized in cells with long-term repopulating potential in the peripheral blood.	[50]
CXCL3	Upregulated in human quiescent HSCs in comparison to proliferating ones; Oct-1 a repressor of CXCL3 is highly expressed in $CD34^+$ HPC.	[51, 52]
CXCL2	Upregulated in human quiescent HSCs in comparison to proliferating ones.	[52]
CXCL12	Constantly expressed in the bone marrow.	[50]
CXCL1	Upregulated in human quiescent HSCs in comparison to proliferating ones.	[52]
CSNK2B	Nothing is known for HSCs, but it is an important factor for bone health indicating secretion by the bone.	[53]
CSNK2A2	q-PCRs showed only CSNK2A2 expression in 1 out of 11 HSCs.	[54]
CHUK	Active when HSC differentiate	[55]
CD40LG	Involved in lymphoproliferation	[56]
CD40	Expressed in the bone marrow.	[57]
CD14	Mesenchymal progenitor cells in humans lack expression of CD14.	[58]
CCL4L2	Oct-1 a repressor of CCL4L2 is highly expressed in $CD34^+$ HPC.	[51]
CCL4	Secreted by the bone marrow.	[50]
CCL21	Secreted by the bone marrow.	[50]

CCL19	Secreted by the bone marrow.	[50]
CCL13	Expressed by surrounding cells.	[59]
CARD11	-	-
CARD10	CARD10 expression accumulates during differentiation.	[60]
BTK	Expressed in all hematopoietic lineage, except T-cells.	[61]
BLNK	Downregulated in aged HSCs.	[62]
BIRC2	-	-
BCL2L1	Important for senescent HSCs.	[63]
BCL2A1	-	-
BCL2	Populations of HSCs show only a few BCL-2 expression cells.	[64]
BCL10	Essential in lymphoid immune cells.	[65]
TRIM25	-	-
TNFRSF14	Involved in mesenchymal stem cells. Not known for hematopoietic stem cells.	-
UBE21	-	-
IL1R1	IL1β signaling is not essential for the normal maintenance of HSCs. Chronic exposure to the ligand inhibits bone marrow hematopoietic stem cell proliferation. IL1R1 loss does not affect HSCs during maintenance or homeostasis.	[66]

Table A.3 Expected hallmarks of HSCs aging matched by the attractor landscape. Mainly described hallmarks of HSC aging are reported and their matched attractor activity. Green indicates that the hallmark is captured by the attractor landscape. Pink indicates that the hallmark is not captured by the attractor pattern.

Hallmark of aging		References
Loss of quiescence/ reduced self-renewal		[62, 67]
Altered DNA damage response		
Myeloid skewing		
Altered cellular polarity		
Altered homing/repopulation abilities		
Increased inflammatory response		
Telomere extension		
Impaired autophagy/metabolic activation		

Figures

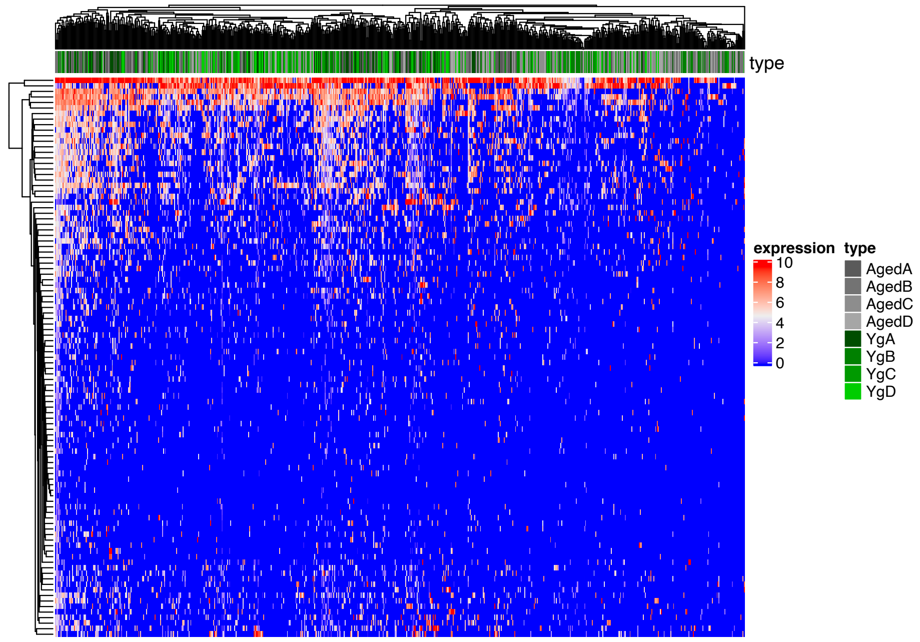

Figure A.1: Clustering of log-normalized gene expression data of NF-κB pathway by individual (eight individuals in total: young A = 19 years old (y.o), B = 40 y.o., C = 21 y.o., D = 37 y.o., aged A = 66 y.o., B = 70 y.o., C = 61 y.o., D = 68 y.o.). Aged Samples are colored in different shades of grey and young in shades of green.

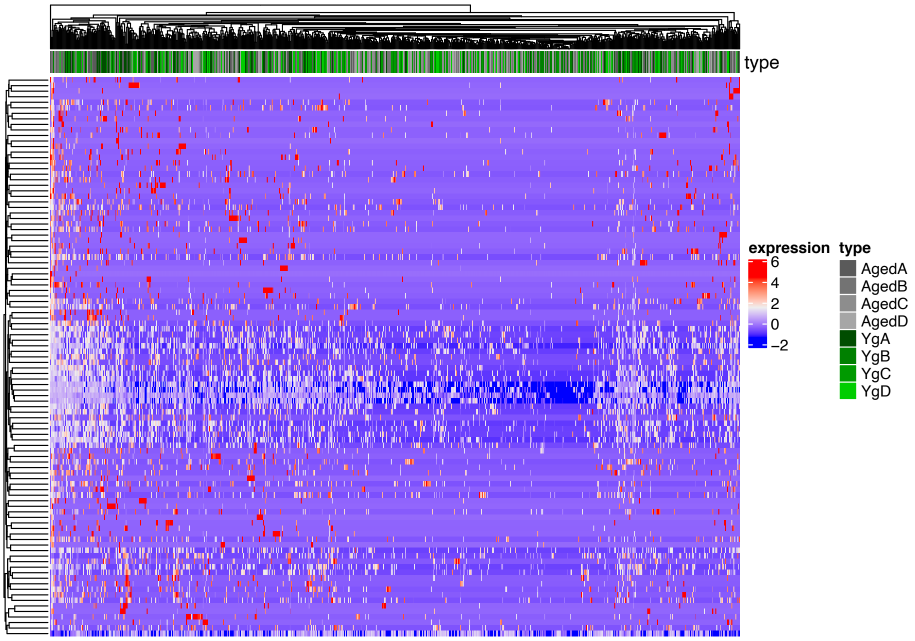

Figure A.2: Clustering log-normalized and z-transformed gene expression data of NF-κB pathway by individual (eight individuals in total: young A = 19 years old (y.o), B = 40 y.o., C = 21 y.o., D = 37 y.o., aged A = 66 y.o., B = 70 y.o., C = 61 y.o., D = 68 y.o.). Aged samples are colored in different shades of grey and young in shades of green.

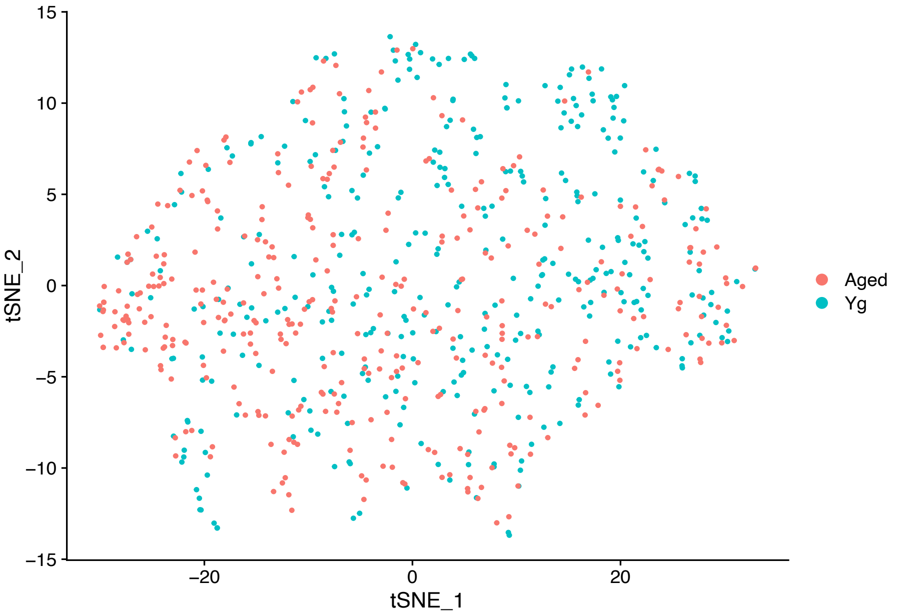

Figure A.3: tSNE plot of gene expression data of NF-κB genes (according to KEGG-DB) using log-normalized expression data. Data was centered and z-transformed before applying tSNE, all using the R-package Seurat. Each dot shows one sample. Aged samples are colored in red, young samples in blue.

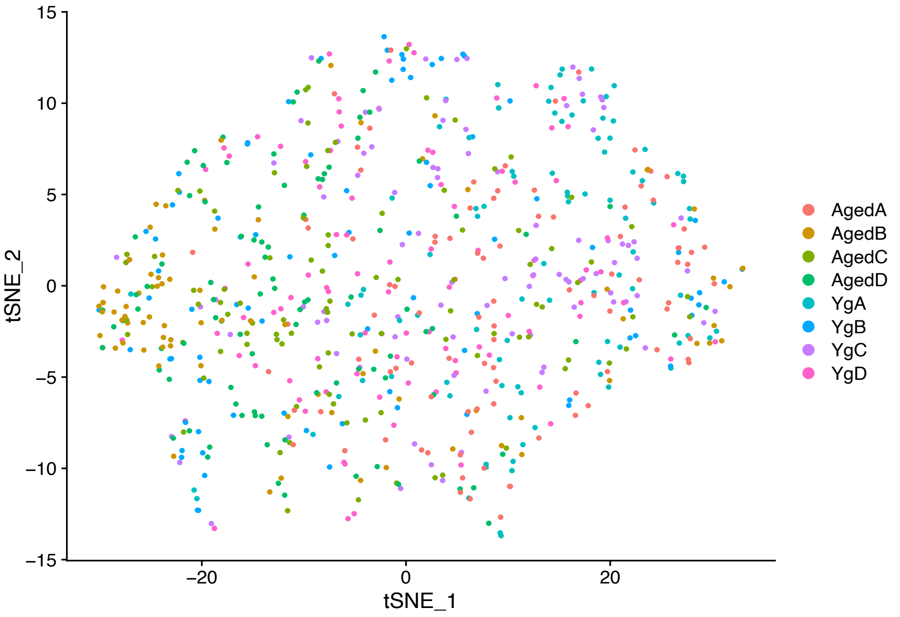

Figure A.4: tSNE plot of gene expression data of NF-κB genes (according to KEGG-DB) using log-normalized expression data. Data was centered and z-transformed before applying tSNE, all using the R-package Seurat. Each dot shows one sample. Samples are colored according to the corresponding individual (eight individuals in total: young A = 19 years old (y.o), B = 40 y.o., C = 21 y.o., D = 37 y.o., aged A = 66 y.o., B = 70 y.o., C = 61 y.o., D = 68 y.o.).

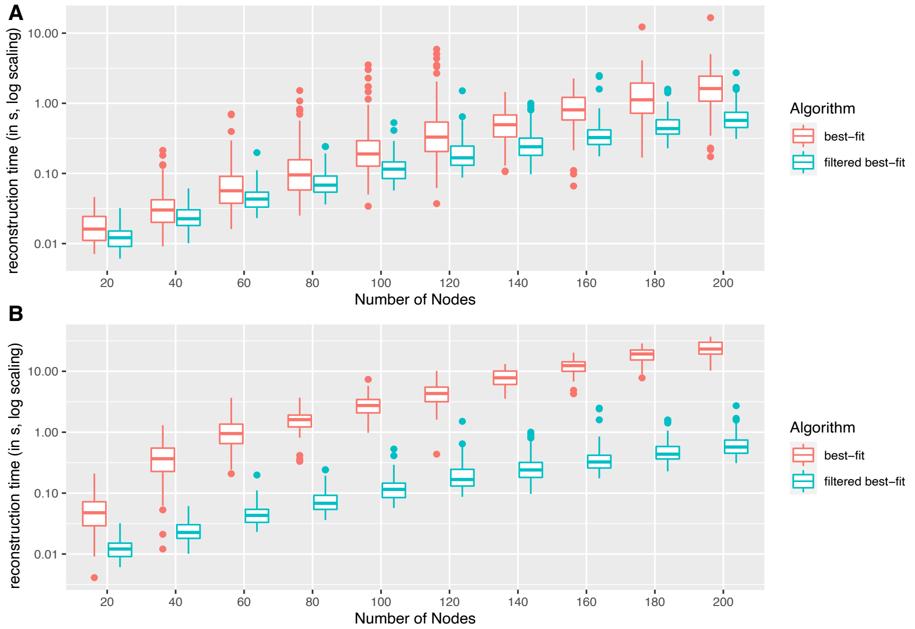

Figure A.5: Runtime of network reconstruction algorithms using *best-fit algorithm* with and without *inferInput* by Maucher et al. [68] as preprocessing step (here named filtered best-fit). Runtime is measured in seconds (y-axis). The x-axis shows the varying network size. 20 time series tuples were measured for each of 100 random networks of each size between 20 and 200 in steps of 20. Simulation was performed for both on non-noisy (A) and noisy (5% noise, B) time-series data.

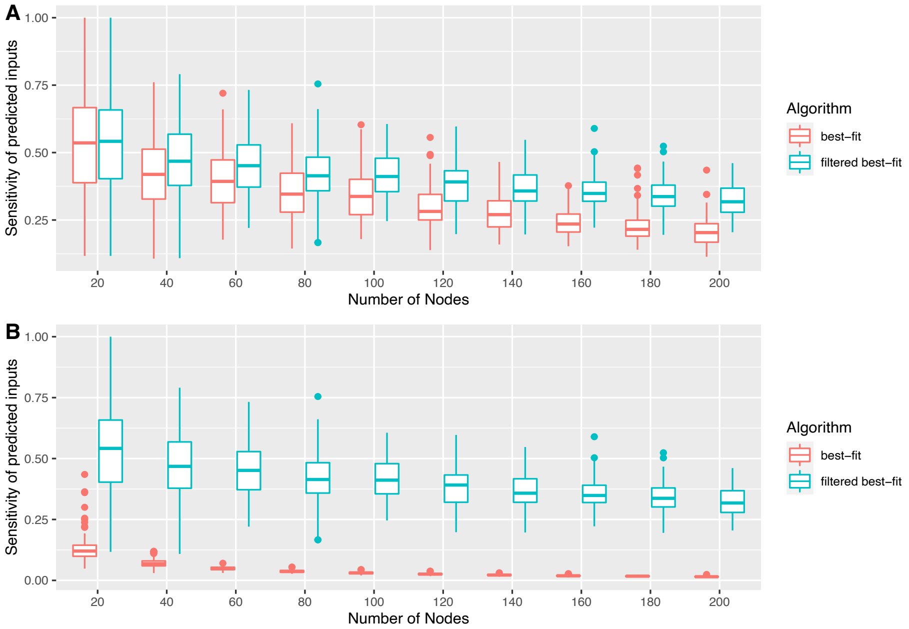

Figure A.6: Boxplots showing the measured sensitivity when predicting the regulatory inputs in each regulatory function for each reconstructed network with the corresponding original network. Figure A shows the results for the reconstruction from data without noise, Figure B for reconstruction from data with 5% noise. The sensitivity distribution is measured over the 100 random networks of each size from 20 to 200 (x-axis), respectively. All reconstructions are based on time- series with 20 time points. Results are measured for the different reconstruction approaches *best-fit* (red) and *filtered best-fit* with *inferInput* by Maucher et al. [68] as preprocessing step (blue).

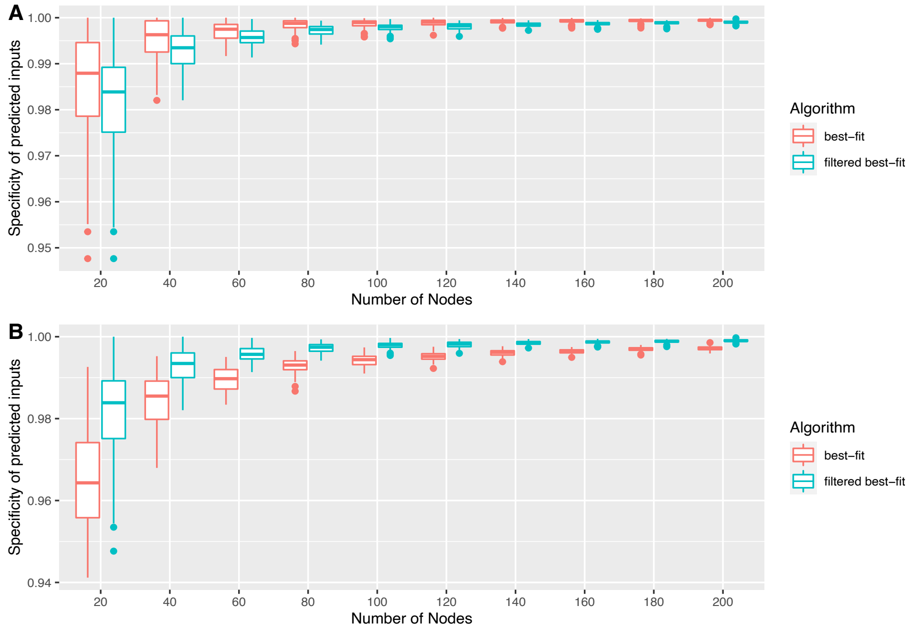

Figure A.7: Boxplots showing the measured specificity when predicting the regulatory inputs in each regulatory function for each reconstructed network with the corresponding original network. Figure A shows the results for the reconstruction from data without noise, Figure B for reconstruction from data with 5% noise. The sensitivity distribution is measured over the 100 random networks of each size from 20 to 200 (x-axis), respectively. All reconstructions are based on time- series with 20 time points. Results are measured for the different reconstruction approaches *best-fit* (red) and *filtered best-fit* with *inferInput* by Maucher et al. [68] as preprocessing step (blue).

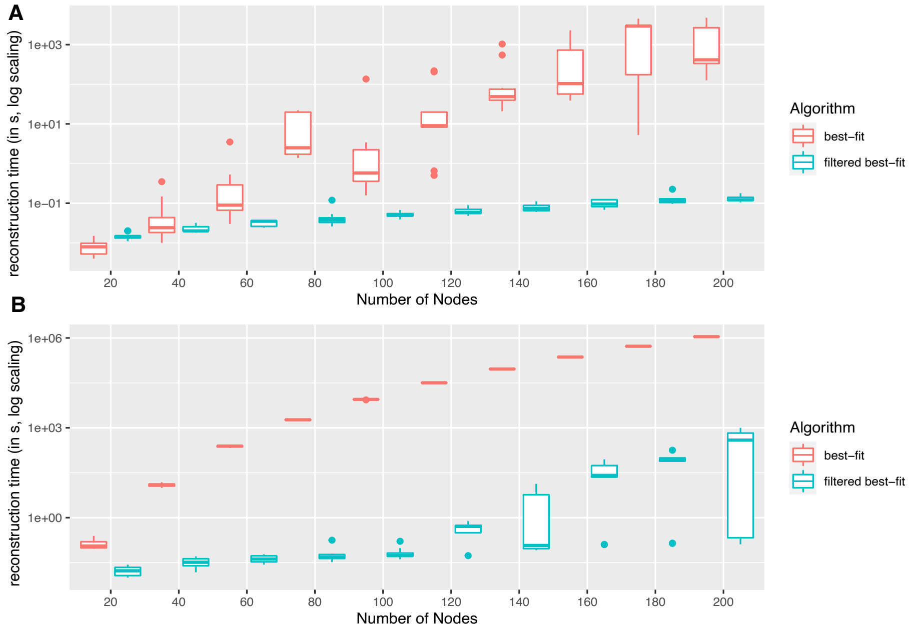

Figure A.8: Runtime of network reconstruction algorithms using best-fit algorithm and filtered best-fit algorithm with *inferInput* by Maucher et al. [68] as preprocessing step. Runtime is measured in seconds (y-axis). Time-scales on the y-axis are shown in logarithmic scaling. The x-axis shows the varying network size. 10 random networks of each size between |V| = 20 and |V| = 200 in steps of 20 were created. Based on these network time-series with |V| + 10 number of time points. Simulation was performed for both on non-noisy (A) and noisy (B) time-series data.

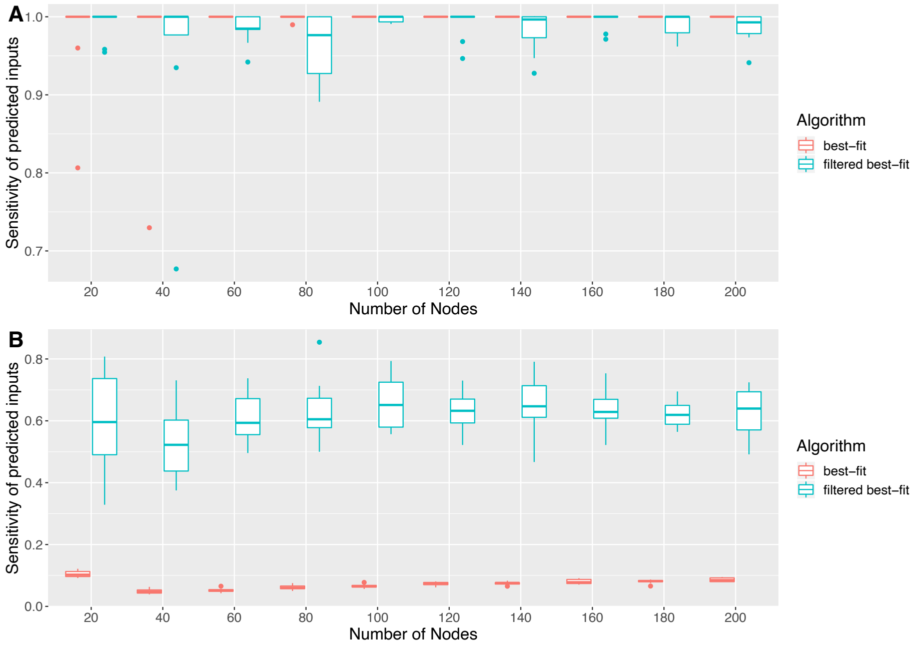

Figure A.9: Boxplots showing the measured sensitivity when predicting the regulatory inputs in each regulatory function for each reconstructed network with the corresponding original network. Figure A shows the results for the reconstruction from data without noise, Figure B for reconstruction from data with 5% noise. The sensitivity distribution is measured over the 100 random networks of each size from 20 to 200 (x-axis), respectively. Reconstructions are based on time-series with |V| + 10 time points, where |V| refers to the number of regulatory components in the network. Results are measured for the different reconstruction approaches *best-fit* (red) and *filtered best-fit* with *inferInput* by Maucher et al. [68] as preprocessing step (blue).

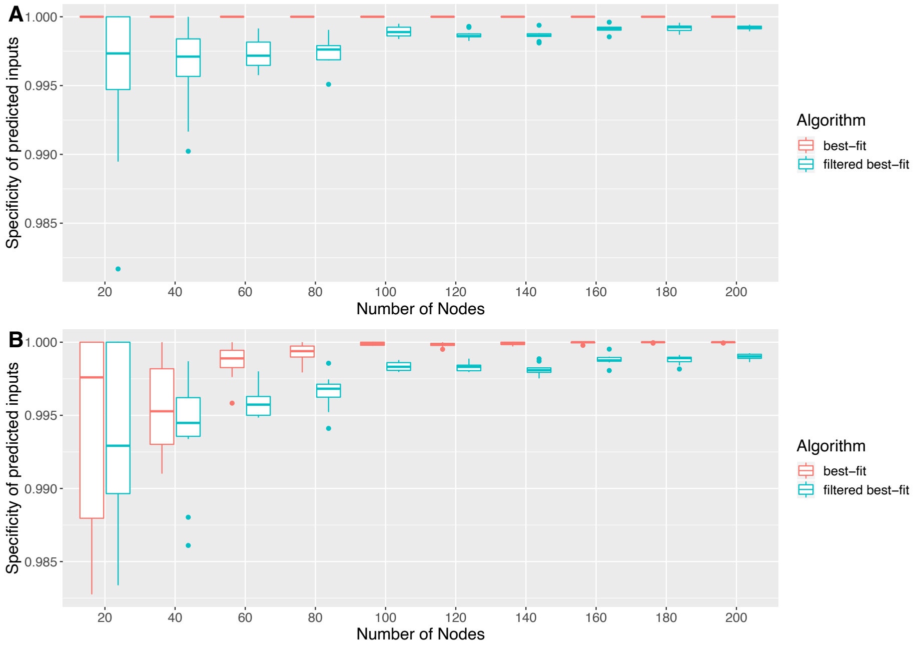

Figure A.10: Boxplots showing the measured specificity when predicting the regulatory inputs in each regulatory function for each reconstructed network with the corresponding original network. Figure A shows the results for the reconstruction from data without noise, Figure B for reconstruction from data with 5% noise. The sensitivity distribution is measured over the 100 random networks of each size from 20 to 200 (x-axis), respectively. Reconstructions are based on time-series with |V| + 10 time points, where |V| refers to the number of regulatory com- ponents in the network. Results are measured for the different reconstruction approaches *best-fit* (red) and *filtered best-fit* with *inferInput* by Maucher et al. [68] as preprocessing step (blue).

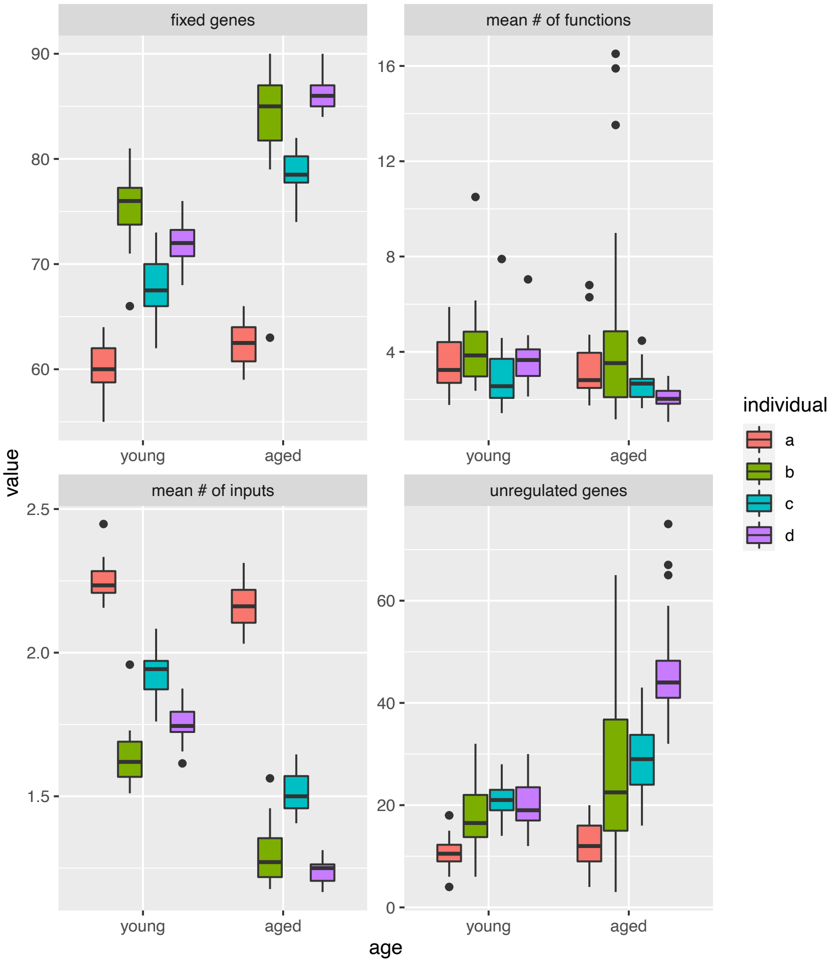

Figure A.11: Static measures per individual. The four properties as described in Section 2.6 for each individual (eight individuals in total: young A = 19 years old. (y.o.), B = 40 y.o., C = 21 y.o., D = 37 y.o., aged A = 66 y.o., B = 70 y.o., C = 61 y.o., D = 68 y.o.).

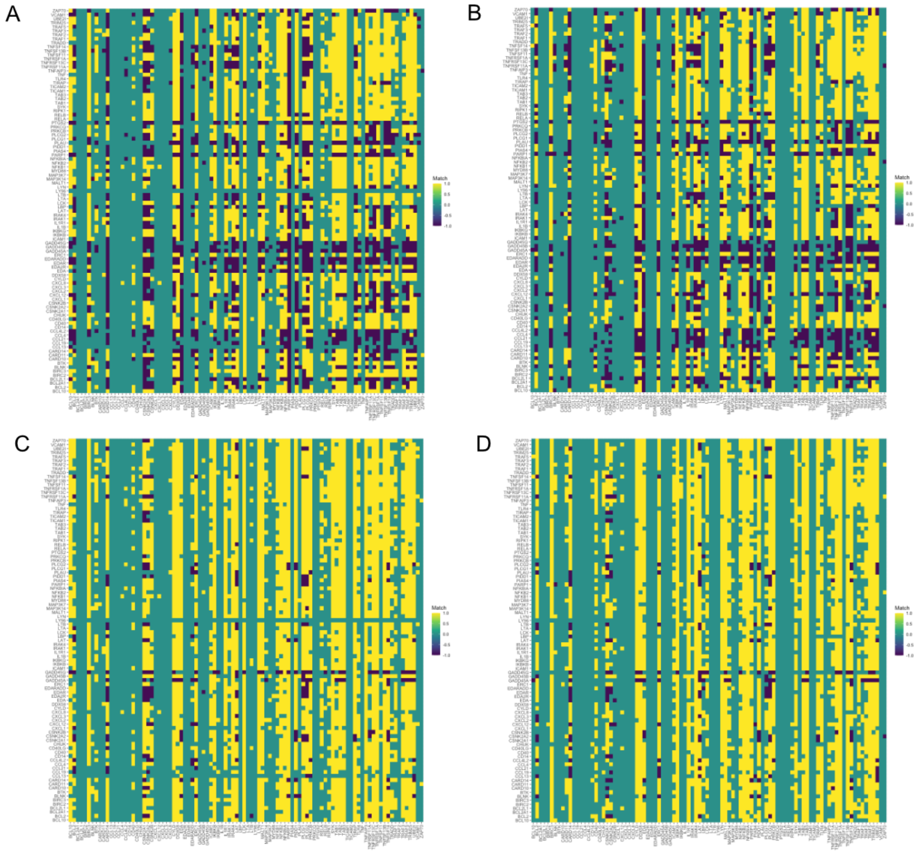

Figure A.12: Comparison of interactions with String DB. (A) shows the matrix of the comparison of all young network interactions to the STRING DB using experimental and database knowledge. (B) shows the matrix of the comparison of all aged network interactions to the STRING DB using experimental and database knowledge. (C) shows the matrix of the comparison of all young network interactions to the STRING DB using experimental, database knowledge, text-mining, and co-expression knowledge. (D) shows the matrix of the comparison of all aged network interactions to the STRING DB using experimental, database knowledge, text-mining, and co-expression knowledge. Yellow indicates matches between Boolean network and STRING DB, green mean no interaction in the Boolean network, and blue indicates a mismatch between Boolean network and STRING DB.

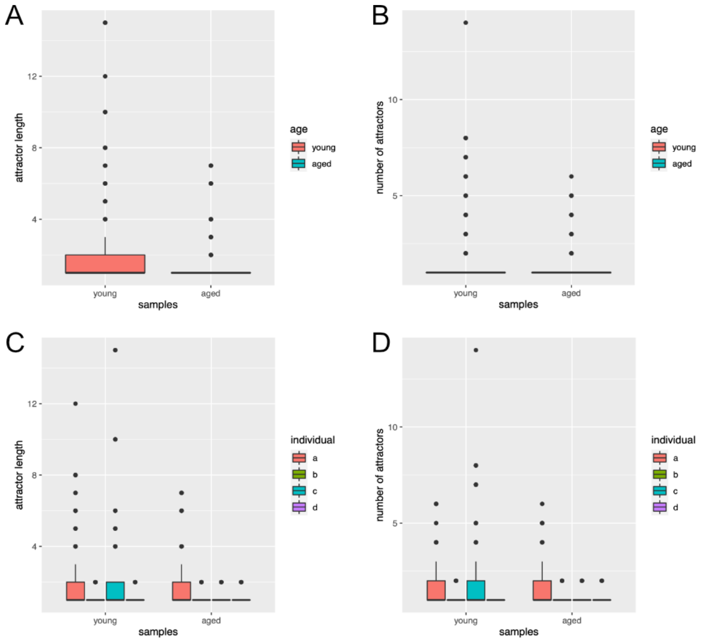

Figure A.13: Boxplots showing attractor properties in different ensembles. The number of attractors of each network within the ensembles was measured and grouped via age (A) and individual (C). The number of attractors found for each network within the ensembles was measured and grouped via age (B) and individual (D). Attractors were simulated exhaustively for all 100 networks in the ensembles representing each individual in 20 repeated runs (1000 random pseudo time tuples per run; eight individuals in total: young A = 19 years old (y.o.), B = 40 y.o., C = 21 y.o., D = 37 y.o., aged A = 66 y.o., B = 70 y.o., C = 61 y.o., D = 68 y.o.).

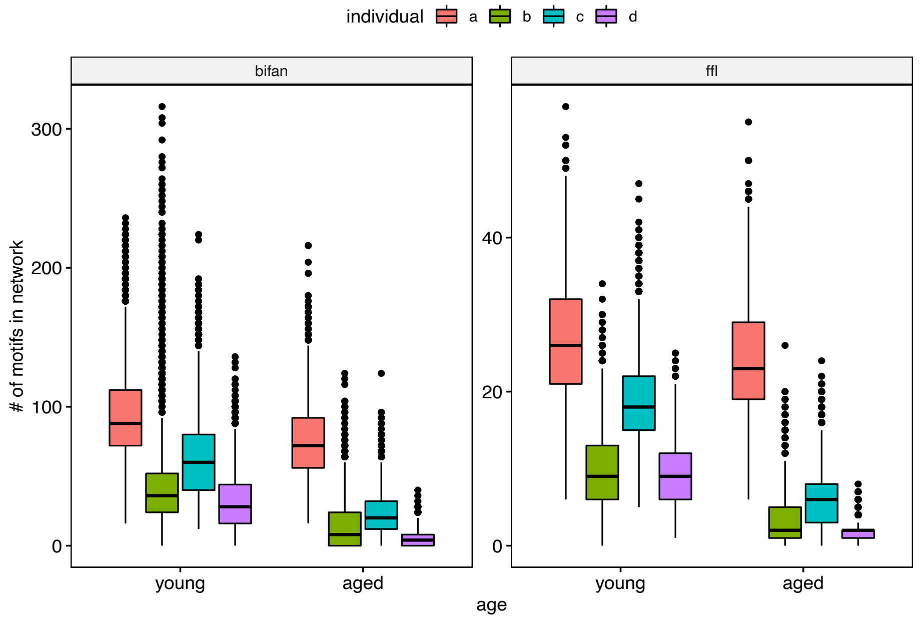

Figure A.14: Boxplots showing the number of bi-fan and feedforward loop motifs measured in the 2000 randomly sampled networks from each individuals' ensemble (100 networks with 20 repetitions). Individuals are encoded as follows: young A = 19 years old (y.o.), B = 40 y.o., C = 21 y.o., D = 37 y.o., aged A = 66 y.o., B = 70 y.o., C = 61 y.o., D = 68 y.o.

References

1. Kowalczyk MS, Tirosh I, Heckl D et al. (2015) Single-cell RNA-seq reveals changes in cell cycle and differentiation programs upon aging of hematopoietic stem cells. Genome Res. 25:1860-1872.
2. Grover A, Sanjuan-Pla A, Thongjuea S et al. (2016) Single-cell RNA sequencing reveals molecular and functional platelet bias of aged haematopoietic stem cells. Nat Commun. 7:11075.
3. Kirschner K, Chandra T, Kiselev V et al. (2017) Proliferation Drives Aging-Related Functional Decline in a Subpopulation of the Hematopoietic Stem Cell Compartment. Cell Rep. 19:1503-1511.

4. Ratliff ML, Garton J, James JA, Webb CF (2020) ARID3a expression in human hematopoietic stem cells is associated with distinct gene patterns in aged individuals. Immun Ageing. 17:1-15.
5. Cuvelier GDE, Rubin TS, Wall DA, Schroeder ML (2016) Long-term outcomes of hematopoietic stem cell transplantation for ZAP70 deficiency. J Clin Immunol. 36:713-724.
6. Otsu M, Steinberg M, Ferrand C et al. (2002) Reconstitution of lymphoid development and function in ZAP-70–deficient mice following gene transfer into bone marrow cells. Blood. 100:1248-1256.
7. Ulyanova T, Scott LM, Priestley GV et al. (2005) VCAM-1 expression in adult hematopoietic and nonhematopoietic cells is controlled by tissue-inductive signals and reflects their developmental origin. Blood. 106:86-94.
8. Lalani AI, Moore CR, Luo C et al. (2015) Myeloid cell TRAF3 regulates immune responses and inhibits inflammation and tumor development in mice. J Immunol. 194:334-348.
9. Amrein MA, Bührer ED, Ruckstuhl C, Höpner S, Riether C, Ochsenbein A (2017) The Traf2 and Nck-Interacting Kinase Is a Conserved Regulator of Self-Renewal in Hematopoietic and Leukemia Stem Cells. Blood.
10. Tesio M, Tang Y, Müdder K et al. (2015) Hematopoietic stem cell quiescence and function are controlled by the CYLD–TRAF2–p38MAPK pathway. J Exp Med. 212:525-538.
11. Baldridge MT, King KY, Goodell MA (2011) Inflammatory signals regulate hematopoietic stem cells. Trends in immunology.
12. Biechonski S, Yassin M, Milyavsky M (2017) DNA-damage response in hematopoietic stem cells: an evolutionary trade-off between blood regeneration and leukemia suppression. Carcinogenesis.
13. Graaf CAD, Kauppi M, Baldwin T et al. (2010) Regulation of hematopoietic stem cells by their mature progeny. Proc Natl Acad Sci U S A. 107:21689-21694.
14. Boyce BF, Xing L (2007) The Rankl/Rank/Opg pathway. Current osteoporosis reports.
15. Schuettpelz L, Link D (2013) Regulation of hematopoietic stem cell activity by inflammation. Front Immunol. 4:204.

16. Smith MA, Culver-Cochran AE, Adelman ER et al. (2020) TNFAIP3 plays a role in aging of the hematopoietic system. Front Immunol. 11:536442.
17. Yamashita M, Passegué E (2019) TNF-α coordinates hematopoietic stem cell survival and myeloid regeneration. Cell Stem Cell. 25:357-372.
18. Lin RC, Zhang Y, Pradhan K, Li L (2020) TICAM2-related pathway mediates neutrophil exhaustion. Sci Rep. 10:14397.
19. Nagai Y, Garrett KP, Ohta S et al. (2006) Toll-like receptors on hematopoietic progenitor cells stimulate innate immune system replenishment. Immunity. 24:801-812.
20. Ibrahim R, Gopal A, Fuller M, Umlandt P, Chang… L (2020) TIRAP drives myelosuppression through an Ifnγ-Hmgb1 axis that disrupts the marrow microenvironment. bioRxiv.
21. Takizawa H, Fritsch K, Kovtonyuk LV et al. (2017) Pathogen-induced TLR4-TRIF innate immune signaling in hematopoietic stem cells promotes proliferation but reduces competitive fitness. Cell Stem Cell. 21:225-240.
22. Takaesu G, Inagaki M, Takubo K et al. (2012) TAK1 (MAP3K7) signaling regulates hematopoietic stem cells through TNF-dependent and-independent mechanisms. PLOS ONE.
23. Roderick JE, Hermance N, Zelic M et al. (2014) Hematopoietic RIPK1 deficiency results in bone marrow failure caused by apoptosis and RIPK3-mediated necroptosis. Proc Natl Acad Sci U S A. 111:14436-14441.
24. Li Z, Fu X, Wu W et al. (2021) Zfp521 is essential for the quiescence and maintenance of adult hematopoietic stem cells under stress. iScience. 24:102039.
25. Zhao C, Xiu Y, Ashton J et al. (2012) Noncanonical NF-κB signaling regulates hematopoietic stem cell self-renewal and microenvironment interactions. Stem Cells. 30:709-718.
26. North TE, Goessling W, Walkley CR et al. (2007) Prostaglandin E2 regulates vertebrate haematopoietic stem cell homeostasis. Nature. 447:1007-1011.
27. Porter RL, Georger MA, Bromberg O et al. (2013) Prostaglandin E2 increases hematopoietic stem cell survival and accelerates hematopoietic recovery after radiation injury. Stem Cells. 31:372-383.

28. Byerly J, Halstead-Nussloch G, Ito K, Katsyv I, Irie H (2016) PRKCQ promotes oncogenic growth and anoikis resistance of a subset of triple-negative breast cancer cells. Breast Cancer Res.
29. Meller N, Elitzur Y, Isakov N (1999) Protein kinase C-θ (PKCθ) distribution analysis in hematopoietic cells: proliferating T cells exhibit high proportions of PKCθ in the particulate fraction. Cell Immunol. 193:185-193.
30. Nakamura-Ishizu A, Takubo K, Kobayashi H, Suzuki-Inoue K, Suda T (2015) CLEC-2 in megakaryocytes is critical for maintenance of hematopoietic stem cells in the bone marrow. J Exp Med. 212:2133-2146.
31. Schnoeder TM, Arreba-Tutusaus P, Mohr J et al. (2016) Myeloid leukemia stem cells depend on phospholipase C gamma 1 (Plcg1) signaling. Blood. 128:1202.
32. Selleri C, Montuori N, Ricci P et al. (2005) Involvement of the urokinase-type plasminogen activator receptor in hematopoietic stem cell mobilization. Blood. 105:2198-2205.
33. Nakagawa MM, Chen H, Rathinam CV (2018) Constitutive activation of NF-κB pathway in hematopoietic stem cells causes loss of quiescence and deregulated transcription factor networks. Front Cell Dev Biol. 6:143.
34. Gil-Kulik P, Dudzińska E, Radzikowska-Büchner E et al. (2020) Different regulation of PARP1, PARP2, PARP3 and TRPM2 genes expression in acute myeloid leukemia cells. BMC Cancer. 20:435.
35. Li X, Erden O, Li L, Ye Q, Wilson A, Du W (2014) Binding to WGR domain by salidroside activates PARP1 and protects hematopoietic stem cells from oxidative stress. Antioxid Redox Signal. 20:1853-1865.
36. Fiedler K, Kokai E, Bresch S, Brunner C (2013) MyD88 is involved in myeloid as well as lymphoid hematopoiesis independent of the presence of a pathogen. Am J Blood Res. 3:124.140.
37. Yáñez A, Murciano C, O'Connor JE, Gozalbo D, Gil ML (2009) Candida albicans triggers proliferation and differentiation of hematopoietic stem and progenitor cells by a MyD88-dependent signaling. Microbes Infect. 11:531-535.
38. Xiu Y, Xue WY, Lambertz A, Leidinger M, Gibson-Corley K, Zhao C (2017) Constitutive activation of NIK impairs the self-

renewal of hematopoietic stem/progenitor cells and induces bone marrow failure. Stem Cells. 35:777-786.
39. McIntyre BAS, Alev C, Tarui H, Jakt LM, Sheng G (2008) Expression profiling of circulating non-red blood cells in embryonic blood. BMC Dev Biol. 8:21.
40. Höpner SS, Raykova A, Radpour R et al. (2021) LIGHT/LTβR signaling regulates self-renewal and differentiation of hematopoietic and leukemia stem cells. Nat Commun.
41. Bogert NV, Furkel J, Din S et al. (2020) A novel approach to genetic engineering of T-cell subsets by hematopoietic stem cell infection with a bicistronic lentivirus. Scientific reports.
42. Gallay P, Heumann D, Le Roy D, Barras C, Glauser MP (1993) Lipopolysaccharide-binding protein as a major plasma protein responsible for endotoxemic shock. Proc Natl Acad Sci U S A. 90:9935-9938.
43. Rhyasen GW, Bolanos L, Fang J et al. (2013) Targeting IRAK1 as a therapeutic approach for myelodysplastic syndrome. Cancer Cell. 24:90-104.
44. Pietras EM, Mirantes-Barbeito C, Fong S et al. (2016) Chronic interleukin-1 exposure drives haematopoietic stem cells towards precocious myeloid differentiation at the expense of self-renewal. Nat Cell Biol. 18:607-618.
45. Liu YF, Zhang SY, Chen YY et al. (2018) ICAM-1 deficiency in the bone marrow niche impairs quiescence and repopulation of hematopoietic stem cells. Stem Cell Reports. 11:258-273.
46. Thalheimer FB, Wingert S, De Giacomo P et al. (2014) Cytokine-regulated GADD45G induces differentiation and lineage selection in hematopoietic stem cells. Stem Cell Reports. 3:34-43.
47. Gupta M, Gupta SK, Balliet AG et al. (2005) Hematopoietic cells from Gadd45a- and Gadd45b-deficient mice are sensitized to genotoxic-stress-induced apoptosis. Oncogene. 24:7170-7179.
48. Himburg HA, Sasine J, Yan X, Kan J, Dressman H, Chute JP (2016) A Molecular Profile of the Endothelial Cell Response to Ionizing Radiation. Radiat Res. 186:141-152.
49. Zhang N-N, Shen S-H, Jiang L-J et al. (2008) RIG-I plays a critical role in negatively regulating granulocytic proliferation. Proc Natl Acad Sci U S A. 105:10553-10558.

50. Mukaida N, Tanabe Y, Baba T (2017) Chemokines as a conductor of bone marrow microenvironment in chronic myeloid leukemia. Int J Mol Sci. 18:1824.
51. Pankratova EV, Stepchenko AG, Krylova ID, Portseva TN, Georgieva SG (2018) The regulatory interplay between Oct-1 isoforms contributes to hematopoiesis and the isoforms imbalance correlates with a malignant transformation of B cells. Oncotarget. 9:29892-29905.
52. Sinclair A, Park L, Shah M et al. (2016) CXCR2 and CXCL4 regulate survival and self-renewal of hematopoietic stem/progenitor cells. Blood. 128:371-383.
53. Chakrabarti S, Ren J, Wu J (2020) Phosvitin derived phospho-peptides show better osteogenic potential than intact phosvitin in MC3T3-E1 osteoblastic cells. Nutrients. 12
54. Ramos CA, Bowman TA, Boles NC et al. (2006) Evidence for diversity in transcriptional profiles of single hematopoietic stem cells. PLoS Genet. 2:e159.
55. Bottero V, Withoff S, Verma IM (2006) NF-kappaB and the regulation of hematopoiesis. Cell Death Differ. 13:785-797.
56. Kuo CY, Long JD, Campo-Fernandez B et al. (2018) Site-specific gene editing of human hematopoietic stem cells for X-linked hyper-IgM syndrome. Cell Rep. 23:2606-2616.
57. Mavroudi I, Papadaki HA (2011) The role of CD40/CD40 ligand interactions in bone marrow granulopoiesis. ScientificWorldJournal. 11:2011-2019.
58. Hackett CH, Flaminio MJ, Fortier LA (2011) Analysis of CD14 expression levels in putative mesenchymal progenitor cells isolated from equine bone marrow. Stem Cells Dev. 20:721-735.
59. Vanbervliet B, Homey B, Durand I et al. (2002) Sequential involvement of CCR2 and CCR6 ligands for immature dendritic cell recruitment: possible role at inflamed epithelial surfaces. Eur J Immunol. 32:231-242.
60. Tooze RM (2013) A replicative self-renewal model for long-lived plasma cells: questioning irreversible cell cycle exit. Front Immunol. 4:460.
61. Nisitani S, Satterthwaite AB, Akashi K, Weissman IL, Witte ON, Wahl MI (2000) Posttranscriptional regulation of Bruton's tyrosine kinase expression in antigen receptor-stimulated splenic B cells. Proc Natl Acad Sci U S A. 97:2737-2742.

62. Li X, Zeng X, Xu Y et al. (2020) Mechanisms and rejuvenation strategies for aged hematopoietic stem cells. J Hematol Oncol. 13:31.
63. Afreen S, Bohler S, Müller A et al. (2020) BCL-XL expression is essential for human erythropoiesis and engraftment of hematopoietic stem cells. Cell Death Dis. 11:8.
64. Orelio C, Dzierzak E (2007) Bcl-2 expression and apoptosis in the regulation of hematopoietic stem cells. Leuk Lymphoma. 48:16-24.
65. Rozmus J, McDonald R, Fung SY et al. (2016) Successful clinical treatment and functional immunological normalization of human MALT1 deficiency following hematopoietic stem cell transplantation. Clin Immunol. 168:1-5.
66. Wang Y, Sun X, Yuan S et al. (2020) Interleukin-1β inhibits normal hematopoietic expansion and promotes acute myeloid leukemia progression via the bone marrow niche. Cytotherapy. 22:127-134.
67. De Haan G, Lazare SS (2018) Aging of hematopoietic stem cells. Blood. 131:479-487.
68. Maucher M, Kracher B, Kühl M, Kestler HA (2011) Inferring Boolean network structure via correlation. Bioinformatics. 27:1529-1536.
